# Brevianamides and Mycophenolic Acid Derivatives from the Deep-Sea-Derived Fungus *Penicillium brevicompactum* DFFSCS025

**DOI:** 10.3390/md15020043

**Published:** 2017-02-17

**Authors:** Xinya Xu, Xiaoyong Zhang, Xuhua Nong, Jie Wang, Shuhua Qi

**Affiliations:** Key Laboratory of Tropical Marine Bio-resources and Ecology, Guangdong Key Laboratory of Marine Materia Medica, RNAM Center for Marine Microbiology, South China Sea Institute of Oceanology, Chinese Academy of Sciences, 164 West Xingang Road, Guangzhou 510301, China; xuxinya@scsio.ac.cn (X.X.); zhangxiaoyong@scsio.ac.cn (X.Z.); xhnong@scsio.ac.cn (X.N.); wangjielangjing@126.com (J.W.)

**Keywords:** *Penicillium brevicompactum*, Brevianamide, Mycochromenic acid derivative, cytotoxicity, antifouling

## Abstract

Four new compounds (**1**–**4**), including two brevianamides and two mycochromenic acid derivatives along with six known compounds were isolated from the deep-sea-derived fungus *Penicillium brevicompactum* DFFSCS025. Their structures were elucidated by spectroscopic analysis. Moreover, the absolute configurations of **1** and **2** were determined by quantum chemical calculations of the electronic circular dichroism (ECD) spectra. Compound **9** showed moderate cytotoxicity against human colon cancer HCT116 cell line with IC_50_ value of 15.6 μM. In addition, **3** and **5** had significant antifouling activity against *Bugula neritina* larval settlement with EC_50_ values of 13.7 and 22.6 μM, respectively. The NMR data of **6**, **8**, and **9** were assigned for the first time.

## 1. Introduction

**Figure 1:**
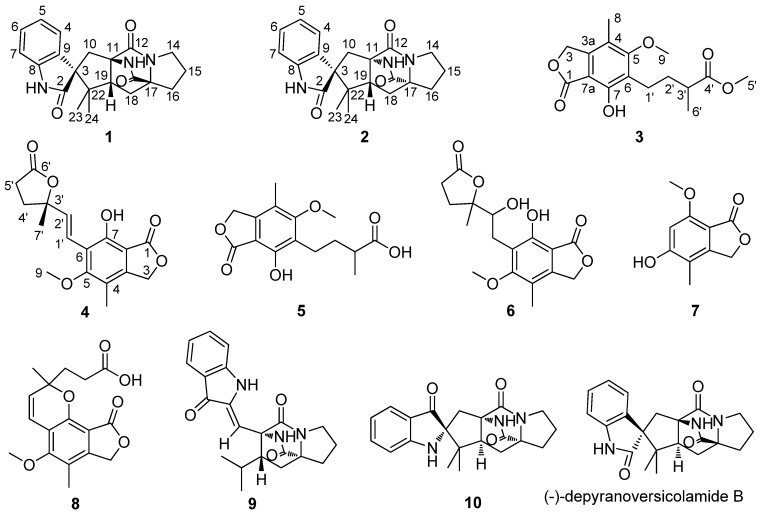
Structures of Compounds **1**–**10**.

Deep-sea-derived microorganisms are new potential resources for discovery of bioactive secondary metabolites [1,2,3]. In our ongoing search for active compounds from marine fungi, four brevianamides and five mycochromenic acid derivatives were obtained from the deep-sea-derived fungus *Penicillium brevicompactum* DFFSCS025. Brevianamides, a class of indole alkaloids, were isolated from *P. brevicompactum* in 1969 for the first time [4]. Their unique bicyclo[2.2.2]diazaoctane skeleton and multiple bio-activities were attractive to scientists. Most of them exhibited anti-bacterial, anti-insect pests and antitubercular potentials [5,6]. Several brevianamides have been totally synthesized [7,8,9]. Mycophenolic acid, a phenyl derivative, was found from *Penicillium* sp. in 1893 for the first time [10]. It was an inhibitor of human inosine 5′-monophosphate dehydrogenase (IMPDH), a target for immunosuppressive chemotherapy [11]. Mycophenolic acid and its derivative mycophenolate mofetil have been used as immunosuppressant drugs in the management of auto-immune disorders since the 1990s [12]. Because of instrument limitations, some brevianamides and mycophenolate acid derivatives are short of reliable spectral data including NMR data [4,10,13]. Herein, we describe the separation, structure elucidation, and bioactivities of Compounds **1**–**10** (Figure 1). The NMR data of **6**, **8**, and **9** were assigned for the first time.

## 2. Results and Discussion

The deep-sea-derived fungal stain DFFSCS025 was inoculated in liquid medium and fermented in standing situation for 32 days at 28 °C. The culture broths were absorbed with XAD-16 resin; meanwhile, mycelium portions were extracted with 80% acetone. The combined extract (24 g from 30 L) was subjected to silica gel column, ODS column, Sephadex LH-20, and purified with semi-preparation HPLC to yield Compounds **1**–**10**.

**Figure 2:**
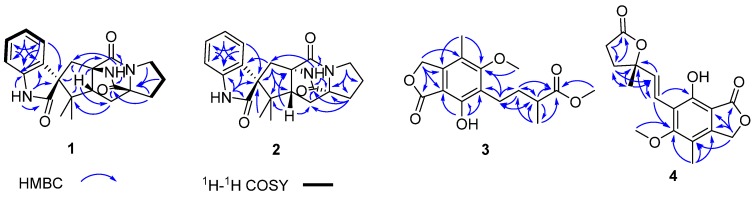
Key HMBC and COSY correlations of **1**–**4**.

**Figure 3:**
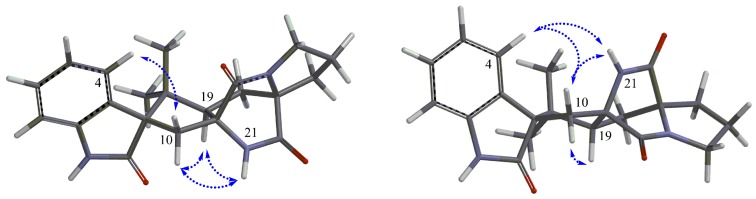
Key NOESY correlations (dashed arrows) of **1** (left) and **2** (right).

**Figure 4:**
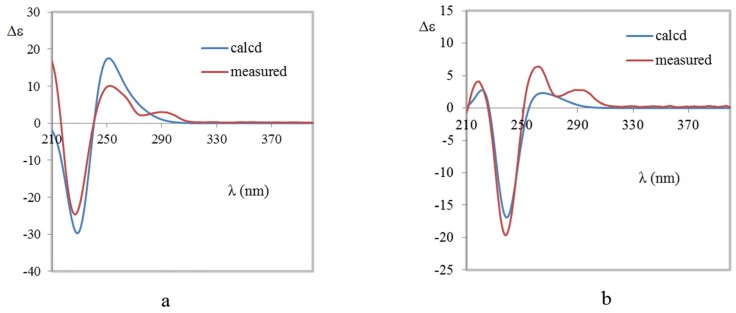
Comparison of the measured and calculated ECD spectra of **1** (**a**) and **2** (**b**). (**a**) ECD spectra of (3*S*, 11*R*, 17*R*, 19*R*)-**1** in MeOH (σ = 0.3 eV, shift = −3 nm); (**b**) ECD spectra of (3*S*, 11*S*, 17*S*, 19*R*)-**2** in MeOH (σ = 0.27 eV, shift = −2 nm).

Brevianamide X (**1**) was obtained as yellowish powder. Its molecular formula of C_21_H_23_N_3_O_3_ was established by HRESIMS (*m/z* 366.1810 [M + H]^+^). The ^1^H NMR spectrum (Table 1) revealed the presence of two methyls at δ_H_ 0.72 (3H, s) and 0.74 (3H, s), four aromatic protons at δ_H_ 6.83 (1H, d, *J* = 7.6 Hz), 6.98 (1H, td, *J* = 0.8, 7.6, 7.6 Hz), 7.20 (1H, td, *J* = 0.9, 7.6, 7.6 Hz), 7.23 (1H, d, *J* = 7.5 Hz), and two exchangeable protons at δ_H_ 9.13 (1H, br s) and 10.36 (1H, br s). The ^13^C NMR spectrum (Table 1) exhibited 21 carbons including two methyls (δ_C_ 20.2, 23.7), five methylenes (δ_C_ 24.8, 29.5, 29.9, 33.6, 43.8), five methines (δ_C_ 55.9, 109.6, 121.5, 126.4, 128.3), and nine quaternary carbons (δ_C_ 45.6, 61.9, 66.1, 68.5, 131.0, 142.8, 169.8, 173.5, 182.8). These NMR data were similar to those of (−)-depyranoversicolamide B (**11**) [7] except the little differences of the chemical shifts of C-3/11/19/20/22. Detailed analysis of 2D NMR spectra revealed that **1** had the same planar structure as (−)-depyranoversicolamide B (**11**) (Figure 2). The relative configuration of **1** was further determined by NOESY spectrum. The NOE correlations between H-10β, H-19, and H-21 established that they were on the same side, while NOE correlation between H-4 and H-10α indicated that they were on the other side (Figure 3). The relative configuration of **1** was therefore proposed to be 3S*, 11R*, 17R*, and 19R*. In order to assign the absolute configuration of **1**, we carried out molecular mechanics calculation using DFT method at B3LYP/6-31G (d) level [14,15]. Furthermore, ECD/TDDFT calculations of all low-energy conformer afforded ECD spectra consistent with the experimental spectrum (Figure 4). The results indicated the 3*S*, 11*R*, 17*R*, 19*R* configuration for **1** on the basis of the relative configuration. Therefore, Compound **1** was inferred to be a diastereomer of (−)-depyranoversicolamide B.

Brevianamide Y (**2**) had same molecular formula of C_21_H_23_N_3_O_3_ as **1** according to HRESIMS (*m/z* 366.1813 [M + H]^+^). The NMR data of **2** showed great similarity to those of **1** with the only obvious difference of the high-field shift of C-19 (from δ_C_ 55.9 in **1** to δ_C_ 50.5 in **2**) (Table 1). Detailed analysis of 2D NMR spectra revealed that **2** had the same planar structure as **1** (Figure 2). In the NOESY spectrum, NOE correlations between H-4, H-10α, H-21, and H-23 suggested they were on the same side, while NOE correlation between H-19 and H-24 disclosed that they were on the other side (Figure 3). The relative configuration of **2** was suggested as 3S*, 11S*, 17S*, and 19R*. The absolute configuration of **2** was also determined by molecular mechanics calculation and quantum chemical computations [14,15]. The calculated ECD curve of 3*S*, 11*S*, 17*S*, and 19*R* was consistent with experimental one (Figure 4). The structure of **2** was confirmed as (3*S*, 11*S*, 17*S*, 19*R*)-brevianamide Y.

The molecular formula of **3** was determined as C_16_H_20_O_6_ by the HRESIMS (*m/z* 331.1164 [M + Na]^+^). The NMR data (Table 2) of **3** was closely resembled with known compound 6-(3-carboxybutyl)-7-hydroxy-5-methoxy-4-methylphthalan-1-one (**5**) [10] except for the presence of an extra methoxy group (δ_H_ 3.68 and δ_C_ 51.6). The HMBC correlations from H_3_-5′ to C-4′ suggested that the methoxy group was linked to the C-4’ of mycophenolic acid skeleton (Figure 2). No specific optical rotation ([α]${}_{D}^{25}$ 0 (*c* 0.1, MeOH)) or circular dichroism spectral data (±0) indicated **3** was isolated as a racemic mixture. We failed to separate the racemic mixture using chiral column by HPLC (CHIRALPAK^®^ IC, Daicel Corporation, Osaka, Japan). Compound **3** was named as 6-(methyl 3-methylbutanoate)-7-hydroxy-5 -methoxy-4-methylphthalan-1-one.

**Figure 5:**
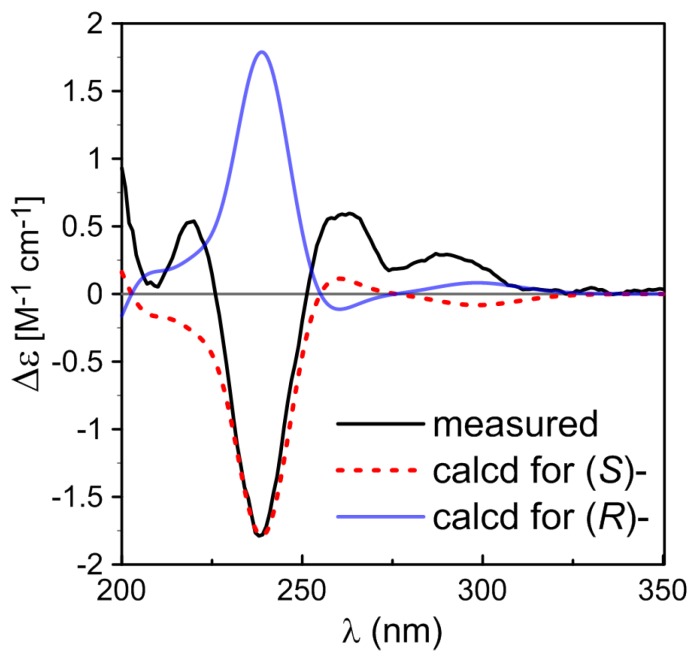
Comparison of the measured ECD spectrum of **4** with B3LYP/TZVP calculated spectra of (*S*)- and (*R*)-**4** in MeOH (σ = 0.20 eV, shift = −18 nm).

The molecular formula of **4** was determined as C_17_H_18_O_6_ by HRESIMS (*m/z* 341.1005 [M + Na]^+^). The similar NMR data (Table 2) of **4** and **3** indicated they had the same benzofuranone skeleton. The down-field shift of C-6 (from δ_C_ 122.5 in **3** to δ_C_ 117.9 in **4**) was induced by the hyperconjugation of a double bond (C-1′, δ_C_ 118.23; C-2′, δ_C_ 137.38) with benzofuranone skeleton, which was confirmed by the HMBC correlations between H-1′/H-2′ and C-6 (Figure 3). The *E*-configured double bond ∆^1′2′^ was determined by the coupling constant value of approximately 16 Hz between H-1′ and H-2′. The NMR data (δ_H_ 2.19, 2.24, 2.56, 2.64; δ_C_ 26.4, 28.8, 34.0, 85.8, 177.0) and HMBC correlations from H-7′ to C-3′, C-4′, from H-4 to C-3′, C-5′, C-6′, C-7′, and H-5′ to C-4′, C-6′ elucidated the presence of a methyl-furanone residue [16,17] attached at C-2′ according to the HMBC correlations from H-1′ to C-3′, and from H-2′ to C-3′. Compound **4** had one chiral center at C-3′. The configuration of **4** was determined by molecular mechanics calculation and quantum chemical computations [14,15]. The (3′*S*)-**4** of calculated ECD curve was consistent with experimental one (Figure 5). Compound **4** was named as (3′*S*)-(*E*)-7-hydroxy-5-methoxy-4-methyl-6-(2-(2-methyl-5-oxotetrahydrofuran-2-yl)vinyl) isobenzofuran-1(3*H*)-one.

Compounds **5**–**10** were identified as 6-(3-carboxybutyl)-7-hydroxy-5-methoxy -4-methylphthalan-1-one [18], 7-hydroxy-6-[2-hydroxy-2-(2-methyl-5-oxotetrahydro-2-furyl) ethyl]-5-methoxy-4-methyl-1-phthalanone [10], 5-hydroxy-7-methoxy-4 –methylphtalide [19], mycochromenic acid [10,20], (−)-brevianamide C [21], and (+)-brevianamide A [22], respectively.

Compounds **1**–**10** were tested for cytotoxicity against human colon cancer HCT116 cell line using sulforhodamine B (SRB) assay. Only Compound **9** exhibited moderate activity with an IC_50_ value of 15.6 µM. In addition, antifouling activities of **1**, **3**, **5**, and **8** were also evaluated in settlement inhibition assays with *Bugula neritina* larvae. The larval settlement bioassay showed that **3** and **5** could strongly inhibit the larvae settlement of *B. neritina* larvae with EC_50_ values of 13.7 and 22.6 μM, respectively, and LC_50_/EC_50_ > 100, while **1** and **8** showed no antilarval activity. Moreover, **1**–**10** were evaluated for their anti-bacterial activities against *Streptococcus mutans* (ATCC35668) and *S. sobrinus* (ATCC33478), anti-fungal activities against *Fusarium oxysporum* f. sp. *cubense* Race 1 and Race 4; however, they did not show any obvious activity at the concentration of 20 µg/mL. 

## 3. Experimental Section

### 3.1. General Experimental Procedure

Optical rotations were measured with an Anton Paar MCP 500 polarimeter (Anton Paar GmbH, Graz, Austria). UV spectra were measured with a Shimadzu UV-2600 UV–vis spectrophotometer (Shimadzu, Kyoto, Japan) in a MeOH solution. Infrared spectra (IR) were recorded on a Shimadzu IRAffinity-1 Fourier transform infrared spectrophotometer (Shimadzu, Kyoto, Japan). ^1^H/^13^C NMR and 2D NMR spectra were recorded on a Bruker AV-500 spectrometer (Bruker, Billerica, MA, USA) with TMS as reference. High-resolution electrospray-ionization (HRESIMS) was performed on a Bruker microTOF-QII mass spectrometer (Bruker, Bremen, Germany). Analysis HPLC was performed on an Angilent 1100 liquid chromatography system (Agilent Technologies, Santa Clara, CA, USA). Semi-preparative reversed-phase HPLC was performed on a Shimadzu LC-20A preparative liquid chromatography (Shimadzu, Kyoto, Japan) with YMC-Pack ODS-A column 250 × 10 mm i.d., S-5 μm × 12 nm, and 250 × 20 mm i.d., S-5 μm × 12 nm. Column chromatography (CC) was performed on silica gel (200–300 mesh, Qingdao Marine Chemical, Qingdao, China), Sephadex LH-20 (GE Healthcare, Barrington, IL, USA), or Rp-18 silica (Pharmacia Co. Ltd., St. Paul, MN, USA).

### 3.2. Fungal Material

The fungal strain DFFSCS025 (GenBank access number JX156371) was isolated from a deep-sea sediment sample collected in the South China Sea, Sansha City (18°5′ N, 118°31′ E; 3928 m depth), Hainan Province, and identified as *Penicillium brevicompactum* by ITS rDNA sequence homology (99% similarity with *P. brevicompactum*). The strain was deposited in the RNAM Center, South China Sea Institute of Oceanology, Chinese Academy of Sciences. 

### 3.3. Fermentation and Extraction

Spores of the fungal strain were inoculated into 1000 mL Erlenmeyer flasks each containing 300 mL of liquid medium (glucose 1%, maltose 2%, monosodium glutamate 1%, KH_2_PO_4_ 0.05%, MgSO_4_·7H_2_O 0.003%, corn steep liquor 0.05%, yeast extract 0.3%, dissolved in sea water, pH 6.5). After 32 days of stationary cultivation at 28 °C, the whole broths (30 L) were filtered through cheesecloth. Sterilized XAD-16 resin (20 g/L) was added to the liquor and shaken at low speed for 30 min to absorb the organic products. The resin was washed with distilled water to remove medium residue then eluted with methanol. The methanol solvent was removed under vacuum to yield a brown residue (18 g). The mycelium portion was smashed and extracted twice with 80% acetone/H_2_O. The acetone soluble fraction was dried in vacuo to yield 6 g of residue. The residues of liquor and mycelium extracts were combined together according to TLC chromatography detecting.

### 3.4. Purification 

The combined extract (24 g) was subjected to silica gel column (500 g) and eluted with CHCl_3_/MeOH (100:0–80:20, *v*/*v*) to yield ten fractions (Fractions 1–10). Fraction 4 (10.5 g) was separated by silica gel column and eluted with CHCl_3_/MeOH to give seven sub-fractions (Fractions 4-1–4-7). Fraction 4-4 (0.9 g) was subjected to Develosil ODS column eluting with a decreasing polarity of MeOH/H_2_O (20:80–70:30) and purified with semi-preparation HPLC (MeOH/H_2_O, 65:35) at the flow rate of 3 mL/min to yield **6** (*t*_R_ 68.2 min, 16.5 mg) and **7** (*t*_R_ 15.3, 2.4 mg). Fraction 6 was isolated with Develosil ODS column eluting with MeOH/H_2_O (15:85–70:30) to obtain six sub-fractions (Fractions 6-1–6-6). Fraction 6-4 was purified by preparatory HPLC (CH_3_CN/H_2_O, 34:66) at the flow rate of 6 mL/min to yield **2** (*t*_R_ 18.4 min, 4.7 mg), **10** (*t*_R_ 25.5 min, 1.7 mg), **9** (*t*_R_ 28.4 min, 2.7 mg), **5** (*t*_R_ 35.8 min, 4.2 mg), and **4** (*t*_R_ 53.4 min, 6.0 mg), respectively. Fraction 6-5 was purified by HPLC (MeOH/H_2_O, 65:35) at the flow rate of 6 mL/min to yield **1** (*t*_R_ 13.6 min, 3.2 mg), **8** (*t*_R_ 15.8 min, 55.3 mg), and **3** (*t*_R_ 27.1 min, 25.5 mg).

Brevianamide X (**1**): yellowish powder; [α]${}_{D}^{25}$ +8.7 (*c* 0.2, MeOH); UV (MeOH) λ_max_ (log ε) 209 (4.44), 253 (3.68), 286 (3.08) nm; CD (MeOH) λ_max_ (∆ε) 227 (‒24.6), 252 (+10.1), 290 (+3.0); ^1^H and ^13^C NMR data, see Table 1; HRESIMS *m/z* 366.1810 [M + H]^+^ (calcd. for 366.1812), 388.1630 [M + Na]^+^ (calcd. for 388.1632).

Brevianamide Y (**2**): yellowish powder; [α]${}_{D}^{25}$ +11.5 (*c* 0.2, MeOH); UV (MeOH) λ_max_ (log ε) 209 (4.64), 252 (3.88), 286 (3.25) nm; CD (MeOH) λ_max_ (∆ε) 207 (‒2.0), 218 (+4.5), 238 (‒21.7), 262 (+7.1), 276 (+2.0), 293 (+3.1); ^1^H and ^13^C NMR data, see Table 1; HRESIMS *m/z* 366.1813 [M + H]^+^ (calcd. for 366.1812), 388.1636 [M + Na]^+^ (calcd. for 388.1632).

*6-(Methyl 3-methylbutanoate)-7-hydroxy-5-methoxy-4-methylphthalan-1-one* (**3**): white powder; [α]${}_{D}^{25}$ 0 (*c* 0.1, MeOH); UV (MeOH) λ_max_ (log ε) 209 (4.67), 286 (3.31), 313 (2.67) nm; CD (MeOH) λ_max_ (∆ε) ±0; ^1^H and ^13^C NMR data, see Table 2; HRESIMS *m/z* 309.1332 [M + H]^+^ (calcd. for 309.1333), 331.1164 [M + Na]^+^ (calcd. for 331.1152). 

*(3′S)-(E)-7-Hydroxy-5-methoxy-4-methyl-6-(2-(2-methyl-5-oxotetrahydrofuran-2-yl)vinyl)isobenzofuran-1(3H)-one* (**4**): white powder; [α]${}_{D}^{25}$ +2.2 (*c* 0.1, MeOH); UV (MeOH) λ_max_ (log ε) 209 (4.05), 244 (4.04), 334 (3.22) nm; CD (MeOH) λ_max_ (∆ε) 210 (+0.05), 220 (+0.54), 238 (‒1.79), 261 (+0.59), 274 (+0.17), 290 (+0.29); ^1^H ^13^C NMR data, see Table 2; HRESIMS *m/z* 341.1005 [M + Na]^+^ (calcd. for 341.0996).

*7-Hydroxy-6-*[2-hydroxy-2-(2-methyl-5-oxotetrahydro-2-furyl)ethyl]*-5-methoxy-4-methyl-1-phthalanone* (**6**): white powder; [α]${}_{D}^{25}$ ‒0.8 (*c* 0.1, MeOH); CD (MeOH) λ_max_ (∆ε) ±0; ^1^H NMR (500 MHz, DMSO-*d*_6_) δ_H_: 5.24 (2H, s, H-3), 2.09 (3H, s, H-8), 3.75 (3H, s, H-9), 2.78 (1H, dd, *J* = 2.0, 13.5 Hz, H-1′α), 2.71 (1H, dd, *J* = 10.1, 13.5 Hz, H-1′β), 3.83 (1H, dd, *J* = 1.9, 9.8 Hz, H-2′), 2.37 (1H, ddd, J = 7.3, 10.0, 12.3 Hz, H-4′α), 1.87 (1H, ddd, *J* = 6.3, 10.3, 12.5 Hz, H-4′β), 2.61 (1H, dd, *J* = 7.3, 10.8 Hz, H-5′α), 2.56 (1H, dd, *J* = 6.4, 10.0 Hz, H-5′β), 1.41 (3H, s, H-7′); ^13^C NMR (125 MHz, DMSO-*d*_6_) δ_C_: 170.23 (C, C-1), 68.85 (CH_2_, C-3), 146.84 (C, C-3a), 116.26 (C, C-4), 163.50 (C, C-5), 120.99 (C, C-6), 154.31 (C, C-7), 107.48 (C, C-7a), 11.58 (CH_3_, C-8), 61.13 (CH_3_, C-9), 26.65 (CH_2_, C-1′), 74.61 (CH, C-2′), 88.65 (C, C-3′), 28.58 (CH_2_, C-4′), 29.35 (CH_2_, C-5′), 177.13 (C, C-6′), 22.53 (CH_3_, C-7′); HRESIMS *m/z* 335.1146 (M − H)^−^ (calcd. for 335.1136).

Mycochromenic acid (**8**): white powder; [α]${}_{D}^{25}$ ‒3.1 (*c* 0.15, MeOH); ^1^H NMR (500 MHz, DMSO-*d*_6_) δ_H_: 5.22 (2H, s, H-3), 2.07 (3H, s, H-8), 3.75 (3H, s, H-9), 6.62 (1H, d, *J* = 10.1 Hz, H-1′), 5.83 (1H, d, *J* = 10.1 Hz, H-2′), 1.93 (2H, m, H-4′), 2.35 (2H, t, *J* = 7.8 Hz, H-5′), 1.40 (3H, s, H-7′); ^13^C NMR (125 MHz, DMSO-*d*_6_) δ_C_: 168.22 (C, C-1), 68.73 (CH_2_, C-3), 148.95 (C, C-3a), 116.98 (C, C-4), 159.36 (C, C-5), 114.70 (C, C-6), 151.03 (C, C-7), 107.95 (C, C-7a), 11.02 (CH_3_, C-8), 62.04 (CH_3_, C-9), 117.52 (CH, C-1′), 130.02 (CH, C-2′), 79.19 (C, C-3′), 35.52 (CH_2_, C-4′), 28.79 (CH_2_, C-5′), 174.54 (C, C-6′), 26.10 (CH_3_, C-7′); ESIMS *m/z* 363 (M + Na)^+^.

(‒)-Brevianamide C (**9**): yellowish powder; [α]${}_{D}^{25}$ −60.4 (*c* 0.2, MeOH); UV (MeOH) λ_max_ (log ε) 202 (4.02), 235 (4.02), 259 (4.13) nm; CD (MeOH) λ_max_ (∆ε) 205 (−16.40), 225 (+10.43), 244 (+1.22), 252 (+1.61), 265 (−2.15), 302 (+0.57); ^1^H NMR (500 MHz, CDCl_3_) δ_H_: 7.70 (1H, d, *J* = 7.5 Hz, H-4), 6.93 (1H, t, *J* = 6.0 Hz, H-5), 7.45 (1H, td, *J* = 6.0, 6.0, 1.0 Hz, H-6), 6.91 (1H, d, *J* = 8.5 Hz, H-7), 5.84 (1H, s, H-10), 3.52 (2H, m, H-14), 2.04 (2H, dt, *J* = 6.5, 6.5, 12.7 Hz, H-15), 1.87 (1H, d, *J* = 15.4 Hz, H-16α), 2.80 (1H, dt, *J* = 6.9, 6.9, 13.4 Hz, H-16β), 1.83 (1H, dd, *J* = 6.0, 13.3 Hz, H-18α), 1.96 (1H, dd, *J* = 10.1, 13.3 Hz, H-18β), 2.46 (1H, m, H-19), 2.13 (1H, m, H-22), 0.85 (3H, d, *J* = 6.9 Hz, H-23), 0.86 (3H, d, *J* = 6.7 Hz, H-24); ^13^C NMR (125 MHz, CDCl_3_) δ_C_: 186.76 (C, C-2), 139.03 (C, C-3), 125.60 (CH, C-4), 120.65 (CH, C-5), 136.80 (CH, C-6), 112.59 (CH, C-7), 154.55 (C, C-8), 121.62 (C, C-9), 104.03 (CH, C-10), 66.89 (C, C-11), 168.83 (C, C-12), 44.48 (CH_2_, C-14), 24.55 (CH_2_, C-15), 29.20 (CH_2_, C-16), 66.21 (C, C-17), 30.83 (CH_2_, C-18), 46.75 (CH, C-19), 172.07 (C, C-20), 27.67 (CH, C-22), 22.16 (CH_3_, C-23), 16.17 (CH_3_, C-24); ESIMS *m/z* 388 (M + Na)^+^.

### 3.5. Computational Methods

Molecular Merck force field (MMFF) and DFT/TDDFT calculations were performed with Spartan′14 software package (Wavefunction Inc., Irvine, CA, USA) and Gaussian09 program package [23], respectively, using default grids and convergence criteria. MMFF conformational search generated low-energy conformers within a 10 kcal/mol energy window were subjected to geometry optimization using the B3LYP/def2-SVP method. Frequency calculations were run with the same method to verify that each optimized conformer was a true minimum and to estimate their relative thermal free energies (∆*G*) at 298.15 K. Energies of the low-energy conformers in MeOH were calculated at the B3LYP/def2-TZVP level. Solvent effects were taken into account by using a polarizable continuum model (PCM). The TDDFT calculations were performed using the hybrid B3LYP [24,25,26] PBE1PBE [27,28] and/or TPSSh [29] functionals, and Ahlrichs’ basis set TZVP (triple zeta valence plus polarization) [30]. The number of excited states per each molecule was 30. The ECD spectra were generated by the program SpecDis [31] using a Gaussian band shape from dipole-length dipolar and rotational strengths. Equilibrium population of each conformer at 298.15 K was calculated from its relative free energies using Boltzmann statistics. The calculated spectra were generated from the low-energy conformers according to the Boltzmann weighting of each conformer in a MeOH solution. 

### 3.6. Cytotoxicity

All compounds were tested for cytotoxicity against HTC116 cell line with SRB method. Briefly, Cytotoxicity assays involving HCT116 cells were performed using sulforhodamine B based on slightly modified protocols [32]. HCT116 cells were maintained in a DMEM medium with 10% fetal bovine serum (FBS) (Life Technologies, Carlsbad, CA, USA). Tested samples were prepared using 10% aqueous DMSO as solvent. The cell suspension was added into 96-well microliter plates in 190 µL at plating densities of 5000 cells/well. One plate was fixed in situ with TCA to represent a no-growth control at the time of drug addition (Day 0). Then, 10 μL of 10% aqueous DMSO was used as control group. After 72 h incubation, the cells were fixed to plastic substratum by the addition of 50 μL of cold 50% aqueous trichloroacetic acid and washed with water after incubation at 4 °C for 30 min. After staining cells with 100 μL of 0.4% sulforhodamine B in 1% aqueous AcOH for 30 min, unbound dye was removed by washing four times with 1% aqueous AcOH. The plates were allowed to dry at room temperature, then the bound dye was solubilized with 200 μL of 10 mM unbuffered Tris base, pH 10. Shaken for 5 min or until the dye was completely solubilized and the optical density was measured at 515 nm using an ELISA plate reader (Bio-Rad, Hercules, CA, USA). The average data were expressed as a percentage, relative to the control. Percentage growth inhibition was calculated as (OD (cells + samples) − OD (Day 0 only cells))/(OD (cells + 10% DMSO) − OD (Day 0 only cells)) = % survival, cytotoxicity = 1 − % survival. (Graphpad Software, Inc., San Diego, CA, USA).

### 3.7. Larval Settlement Assays

Larval culture and larval settlement assays matched the method reported in the literature [33]. Briefly, the stock solution of tested samples in DMSO was diluted with autoclaved filtered sea water (FSW) to concentrations ranging from 3.125 to 100 µg/mL. About 20 competent larvae were added to each well in 1 mL of the test solution. Wells containing only FSW with DMSO served as the controls. The plates were incubated at 27 °C for 1 h for *B. neritina* and 24 h for *B. amphitrite*. The percentage of larval settlement was determined by counting the settled, live individuals under a dissecting microscope and expressing the result as a proportion of the total number of larvae in the well. EC_50_ (inhibits 50% of larvae settlement in comparison with the control) and LC_50_ (lethal concentration, 50%) were calculated by using the Excel software program.

## 4. Conclusions

In conclusion, four new compounds (**1**–**4**), include two brevianamides and two mycochromenic acid derivatives along with six known compounds were isolated from the deep-sea-derived fungus *Penicillium brevicompactum* DFFSCS025. Their structures were elucidated by spectroscopic analysis and quantum chemical computations. Compound **9** showed moderate cytotoxicity against human colon cancer HCT116 cell line with IC_50_ values of 15.6 µM. Compounds **3** and **5** had significant antifouling activity against *Bugula neritina* larval settlement with EC_50_ values of 13.7 and 22.6 μM, respectively. The NMR data of **6**, **8**, and **9** were assigned for the first time.

## Figures and Tables

**Table 1:** ^1^H NMR data (500 MHz) and ^13^C NMR data (125 MHz) of **1** and **2** in DMSO-*d*_6_.

No.	1		2	
	δ_C_	δ_H_	δ_C_	δ_H_
1-N*H*	-	10.36 s	-	10.31 s
2	182.8 C		182.3 C	
3	61.9 C		62.6 C	
4	126.4 CH	7.23, d (7.5)	126.8 CH	7.43, d (7.5)
5	121.5 CH	6.98, dd (7.5, 7.6)	121.3 CH	6.99, dd (7.5, 7.6)
6	128.3 CH	7.20, dd (7.6, 7.6)	128.6 CH	7.20, dd (7.6, 7.6)
7	109.6 CH	6.83, d (7.6)	109.5 CH	6.81, d (7.6)
8	142.8 C		142.9 C	
9	131.0 C		130.2 C	
10	33.6 CH_2_	2.20, d (14.2)	34.1 CH_2_	2.14, d (15.2)
		2.86, d (14.2)		2.83, d (15.2)
11	66.1 C		67.6 C	
12	169.8 C		169.5 C	
14	43.8 CH_2_	3.41, m	43.7 CH_2_	3.30, m
15	24.8 CH_2_	1.80, m	24.9 CH_2_	1.83, m
		1.99, m		2.00, dd (5.9, 12.1)
16	29.5 CH_2_	2.50, overlapped	28.9 CH_2_	1.79, m
				2.47, dd (6.4, 12.1)
17	68.5 C		69.1 C	
18	29.9 CH_2_	1.78, dd (8.2, 12.8)	28.4 CH_2_	1.79, m
		1.93, dd (10.4, 12.9)		2.47, dd (6.4, 12.1)
19	55.9 CH	3.23, dd (8.3, 10.1)	50.5 CH	3.18, dd (5.0, 10.0)
20	173.5 C		173.0 C	
21-N*H*	-	9.13, s	-	8.81, s
22	45.6 C		47.3 C	
23	20.2 CH_3_	0.74, s	20.9 CH_3_	1.00, s
24	23.7 CH_3_	0.72, s	23.5 CH_3_	0.69, s

**Table 2:** ^1^H NMR data (500 MHz) and ^13^C NMR data (125 MHz) of **3** and **4**.

No.	3 ^a^		4 ^b^	
	δ_C_	δ_H_	δ_C_	δ_H_
1	172.9 C		170.6 C	
3	70.0 CH_2_	5.18, s	69.2 CH_2_	5.30, s
3a	144.1 C		147.5 C	
4	116.7 C		117.0 C	
5	163.8 C		162.8 C	
6	122.5 C		117.9 C	
7	153.7 C		153.5 C	
7a	106.3 C		107.8 C	
8	11.6 CH_3_	2.13, s	11.3 CH_3_	2.09, s
9	61.1 CH_3_	3.77, s	60.6 CH_3_	3.67, s
1′	21.3 CH_2_	2.66, m	118.2 CH	6.63, d (16.5)
2′	33.1 CH_2_	1.90, m	137.4 CH	6.76, d (16.1)
		1.67, m		
3′	39.4 CH	2.50, dq (6.9, 6.9)	85.8 C	
4′	177.0 C		34.0 CH_2_	2.24, m
				2.19, m
5′	51.6 CH_3_	3.68, s	28.8 CH_2_	2.64, m
				2.56, m
6′	17.1 CH_3_	1.21 d (1.2)	177.0 C	
7′			26.4 CH_3_	1.55, s

^a^ CDCl_3_ used as solvent. ^b^ DMSO-*d*_6_ used as solvent.

## Supplementary Materials

Available online: www.mdpi.com/1660-3397/15/2/43/s1. ^1^H, ^13^C, and 2D NMR spectra and HRESIMS of Compounds **1**–**4** and known Compounds **6**, **8**, and **9**. HPLC spectra of Compounds **3** and **5**. Chiral HPLC spectra of Compounds **3** and **6**.
