# Supplementary material for: Brevianamides and Mycophenolic Acid Derivatives from the Deep-Sea-Derived Fungus Penicillium brevicompactum DFFSCS025
Source: Mar Drugs. 2017 Feb 17;15(2):43. doi: 10.3390/md15020043 (PMC5334623; doi:10.3390/md15020043)
Supplement: Supplementary file 1 [file marinedrugs-15-00043-s001.pdf]

Supporting Information

Brevianamides and mycophenolic acid derivatives from the Deep-Sea-Derived Fungus *Penicillium brevicompactum* DFFSCS025

Xinya Xu,[1] Xiaoyong Zhang,[1] Xuhua Nong,[1] Jie Wang[1], and Shuhua Qi[*,1]

[1] Key Laboratory of Tropical Marine Bio-resources and Ecology, Guangdong Key Laboratory of Marine Materia Medica, RNAM Center for Marine Microbiology, South China Sea Institute of Oceanology, Chinese Academy of Sciences, 164 West Xingang Road, Guangzhou 510301 Guangdong, China; E-Mails: xuxinya@scsio.ac.cn (X.-Y.X.); zhangxiaoyong@scsio.ac.cn (X.-Y.Z.); xhnong@scsio.ac.cn (X.-H.N.); wangjielangjing@126.com (J.W.); shuhuaqi@scsio.ac.cn (S.-H. Q)

***** Author to whom correspondence should be addressed; E-Mail: shuhuaqi@scsio.ac.cn; Tel.: +86-20-8902-2112; Fax: +86-20-8445-8964.

List of contents

Figure S1. ^{1}H NMR spectrum for compound **1**.

Figure S2. ^{13}C NMR spectrum for compound **1**.

Figure S3. HSQC spectrum for compound **1**.

Figure S4. HMBC spectrum for compound **1**.

Figure S5. ^{1}H-^{1}H COSY spectrum for compound **1**.

Figure S6. NOESY spectrum for compound **1**.

Figure S7. HR-ESIMS spectrum for compound **1**.

Figure S8. ^{1}H NMR spectrum for compound **2**.

Figure S9. ^{13}C NMR spectrum for compound **2**.

Figure S10. HSQC spectrum for compound **2**.

Figure S11. HMBC spectrum for compound **2**.

Figure S12. NOESY spectrum for compound **2**.

Figure S13. HR-ESIMS spectrum for compound **2**.

Figure S14. ^{1}H NMR spectrum for compound **3**.

Figure S15. ^{13}C NMR spectrum for compound **3**.

Figure S16. HSQC spectrum for compound **3**.

Figure S17. HMBC spectrum for compound **3**.

Figure S18. HR-ESIMS spectrum for compound **3**.

Figure S19. ^{1}H NMR spectrum for compound **4**.

Figure S20. ^{13}C NMR spectrum for compound **4**.

Figure S21. HSQC spectrum for compound **4**.

Figure S22. HMBC spectrum for compound **4**.

Figure S23. HR-ESIMS spectrum for compound **4**.

Figure S24. ^{1}H NMR spectrum for compound **6**.

Figure S25. ^{13}C NMR spectrum for compound **6**.

Figure S26. HSQC spectrum for compound **6**.

Figure S27. HMBC spectrum for compound **6**.

Figure S28. HR-ESIMS spectrum for compound **6**.

Figure S29. Original CD spectrum for compound **6**.

Figure S30. ^{1}H NMR spectrum for compound **8**.

Figure S31. ^{13}C NMR spectrum for compound **8**.

Figure S32. HMBC spectrum for compound **8**.

Figure S33. ESIMS spectrum for compound **8**.

Figure S34. ^{1}H NMR spectrum for compound **9**.

Figure S35. ^{13}C NMR spectrum for compound **9**.

Figure S36. HSQC spectrum for compound **9**.

Figure S37. ^{1}H-^{1}H COSY spectrum for compound **9**.

Figure S38. HMBC spectrum for compound **9**.

Figure S39. NOESY spectrum for compound **9**.

Figure S40. HR-ESIMS spectrum for compound **9**.

Figure S41. HPLC chromatogram for compounds **3**.

Figure S42. HPLC chromatogram for compound **5**.

Figure S43. Chiral HPLC chromatogram spectrum for compound **3**.

Figure S44. Chiral HPLC chromatogram spectrum for compound **6**.

Figure S1. ^{1}H NMR spectrum for compound **1**

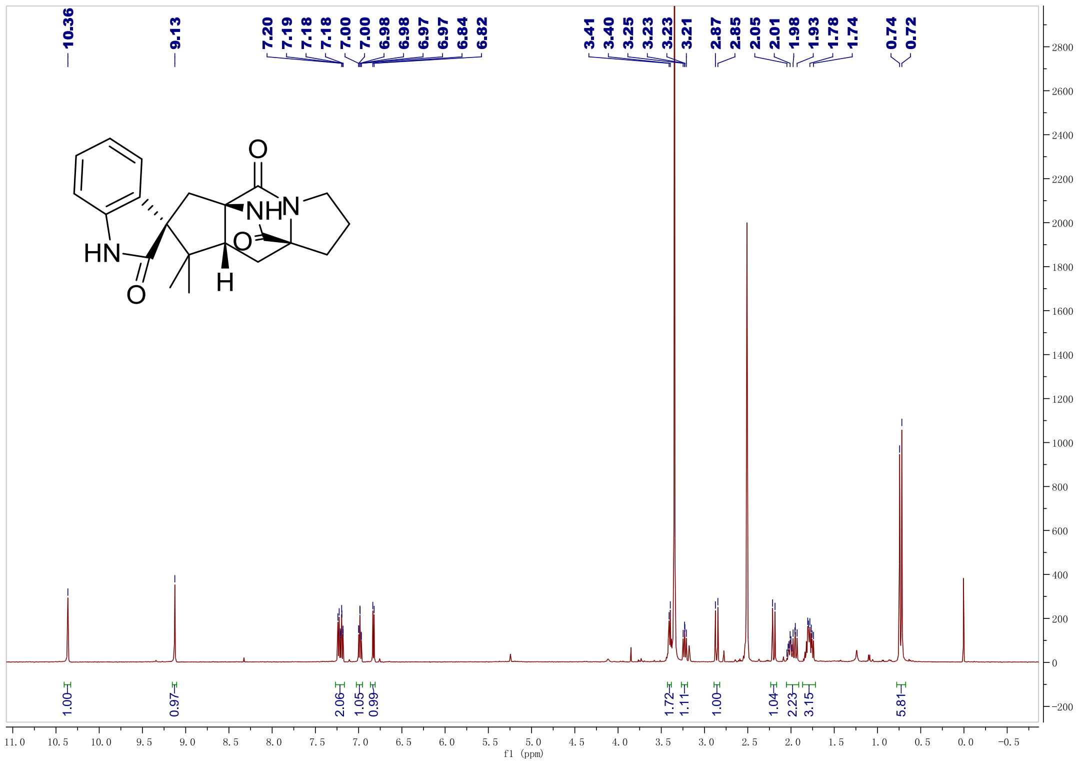

Figure S2. ^{13}C NMR spectrum for compound **1**

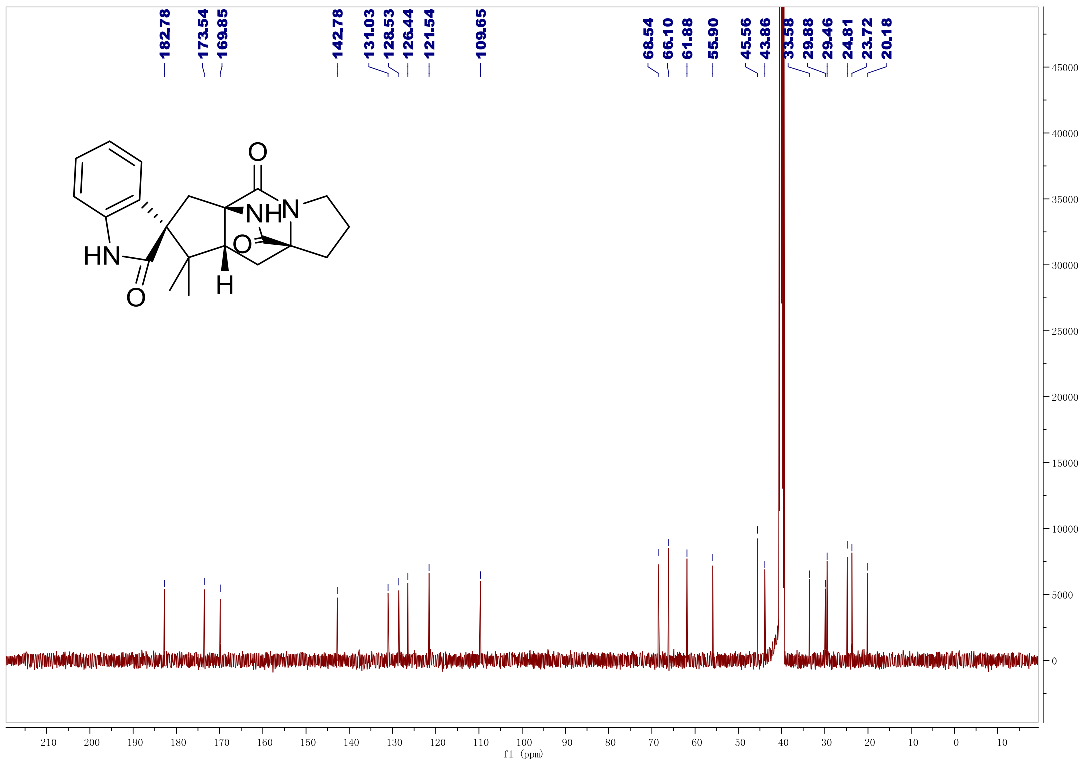

Figure S3. HSQC spectrum for compound **1**

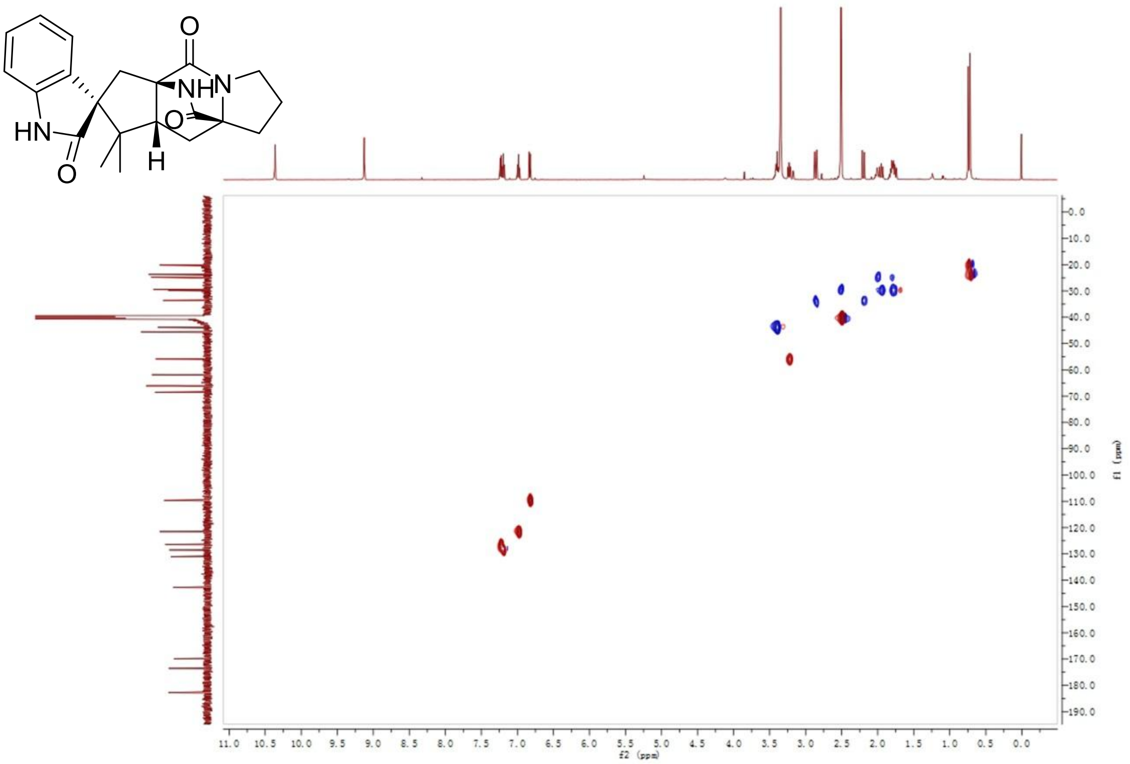

Figure S4. HMBC spectrum for compound **1**

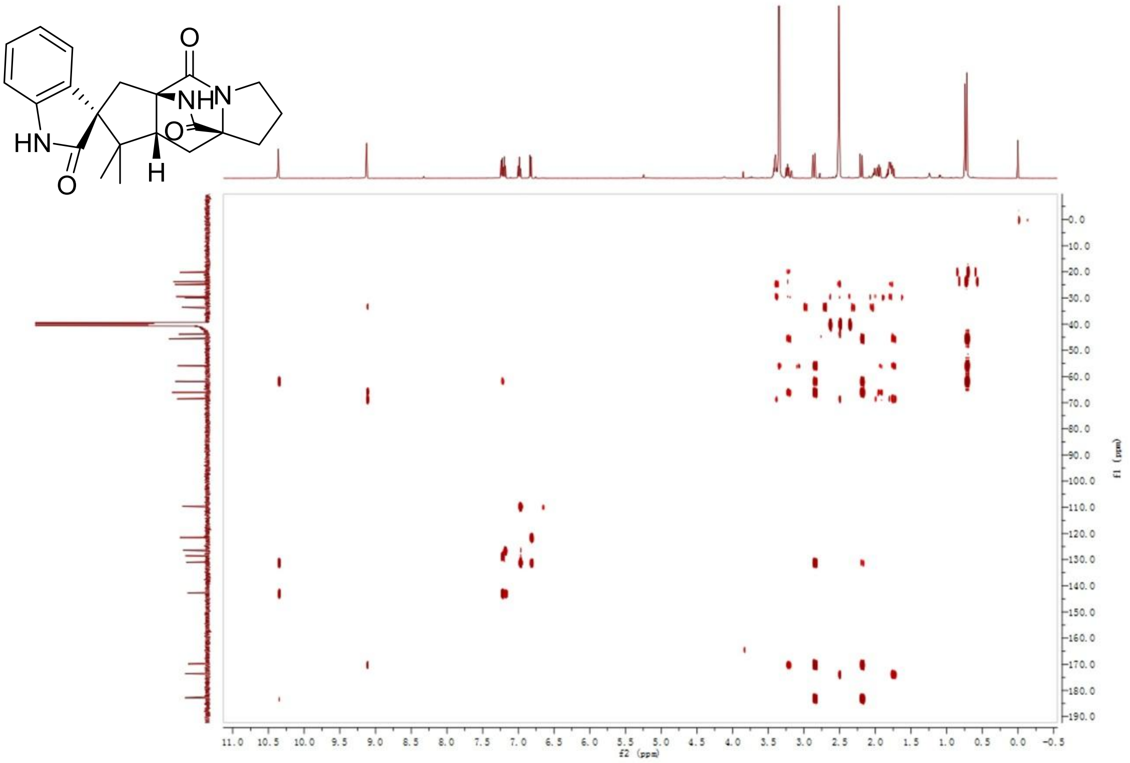

Figure S5. ^{1}H-^{1}H COSY spectrum for compound **1**

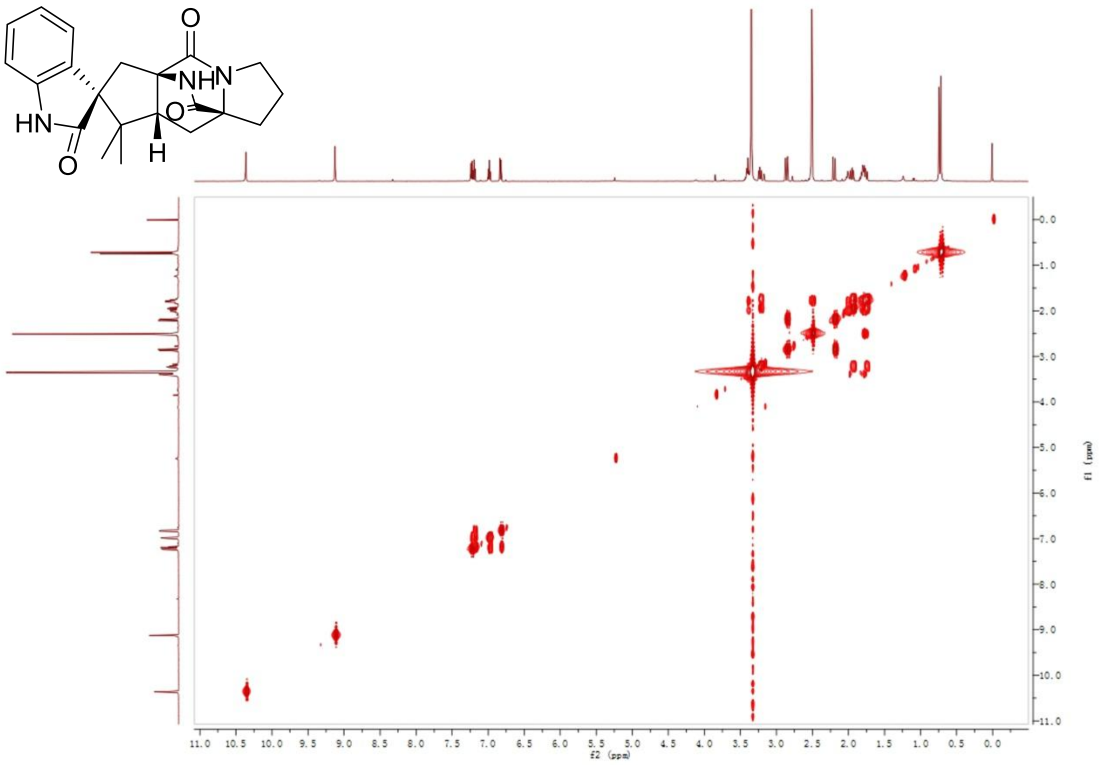

Figure S6. NOESY spectrum for compound **1**

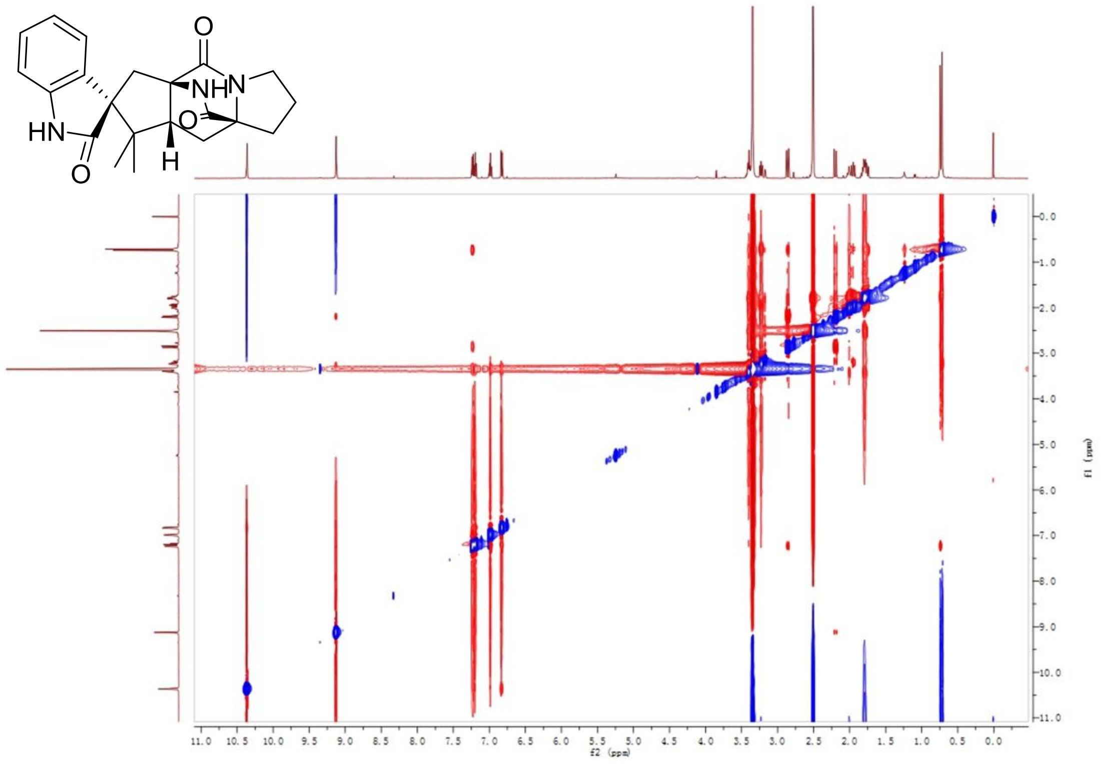

Figure S7. HR-ESIMS spectrum for compound **1**

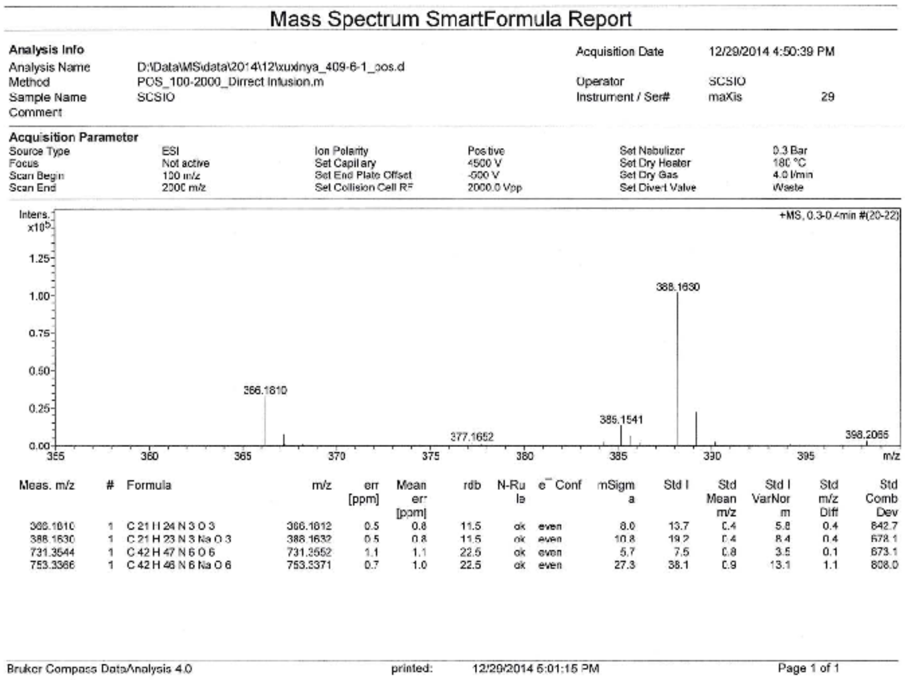

Mass Spectrum SmartFormula Report

**Analysis Info**		Acquisition Date	12/29/2014 4:50:39 PM
Analysis Name	D:\Data\MS\data\2014\12\xuxinya_409-6-1_pos.d		
Method	POS_100-2000_Dirrect Infusion.m	Operator	SCSIO
Sample Name	SCSIO	Instrument / Ser#	maXis 29
Comment			

**Acquisition Parameter**					
Source Type	ESI	Ion Polarity	Positive	Set Nebulizer	0.3 Bar
Focus	Not active	Set Capillary	4500 V	Set Dry Heater	180 °C
Scan Begin	100 m/z	Set End Plate Offset	-500 V	Set Dry Gas	4.0 l/min
Scan End	2000 m/z	Set Collision Cell RF	2000.0 Vpp	Set Divert Valve	Waste

Meas. m/z	#	Formula	m/z	err [ppm]	Mean err [ppm]	rdb	N-Rule	e⁻ Conf	mSigma	Std I	Std Mean m/z	Std I VarNorm	Std m/z Diff	Std Comb Dev
366.1810	1	C 21 H 24 N 3 O 3	366.1812	0.5	0.8	11.5	ok	even	8.0	13.7	0.4	5.8	0.4	842.7
388.1630	1	C 21 H 23 N 3 Na O 3	388.1632	0.5	0.8	11.5	ok	even	10.8	19.2	0.4	8.4	0.4	678.1
731.3544	1	C 42 H 47 N 6 O 6	731.3552	1.1	1.1	22.5	ok	even	5.7	7.5	0.8	3.5	0.1	673.1
753.3366	1	C 42 H 46 N 6 Na O 6	753.3371	0.7	1.0	22.5	ok	even	27.3	38.1	0.9	13.1	1.1	808.0

Bruker Compass DataAnalysis 4.0 printed: 12/29/2014 5:01:15 PM Page 1 of 1

Figure S8. ^{1}H NMR spectrum for compound **2**

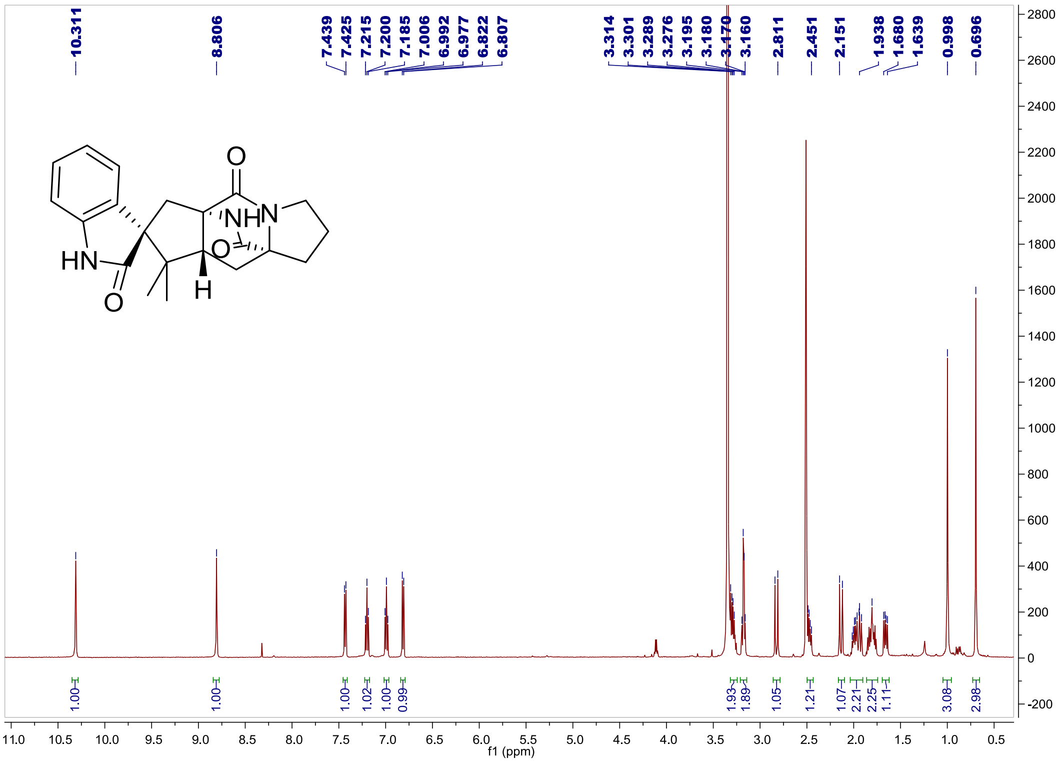

Figure S9. ^{13}C NMR spectrum for compound **2**

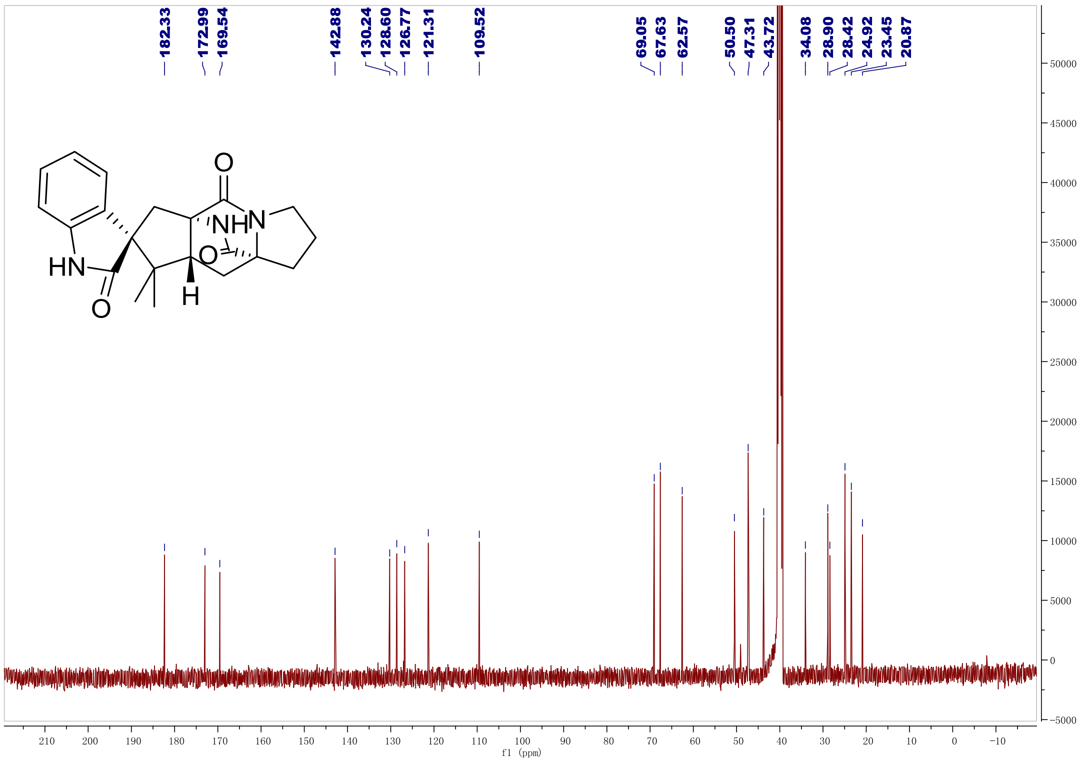

Figure S10. HSQC spectrum for compound **2**

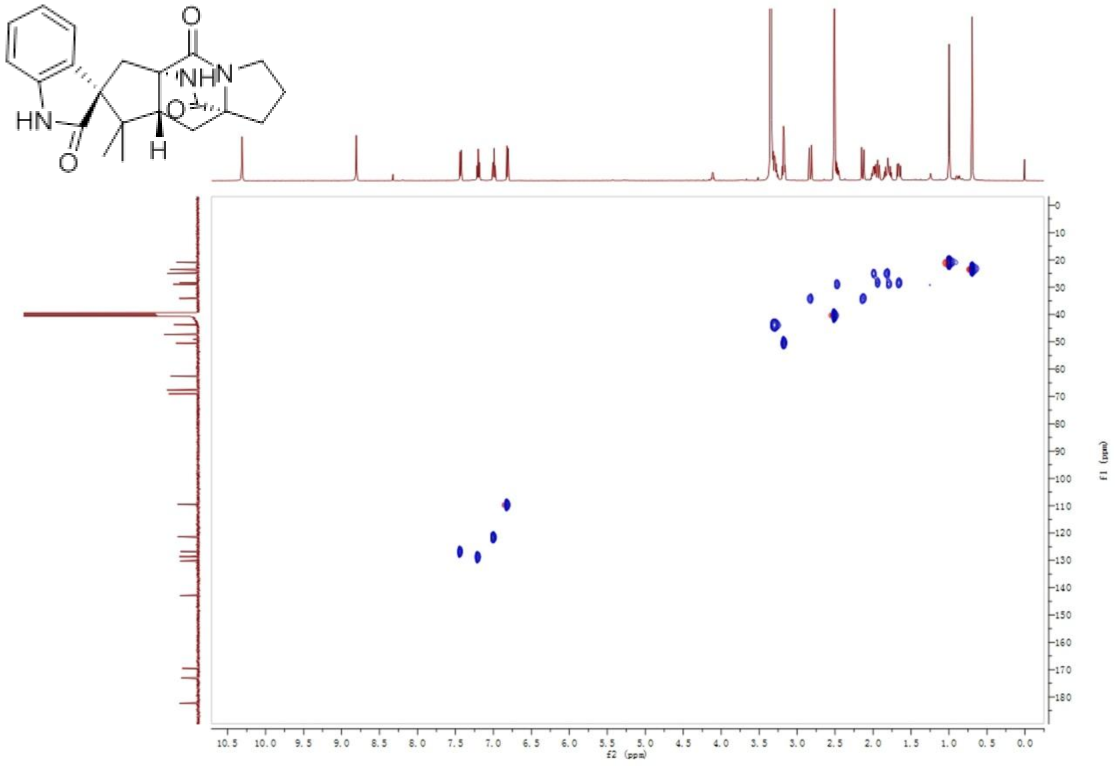

Figure S11. HMBC spectrum for compound **2**

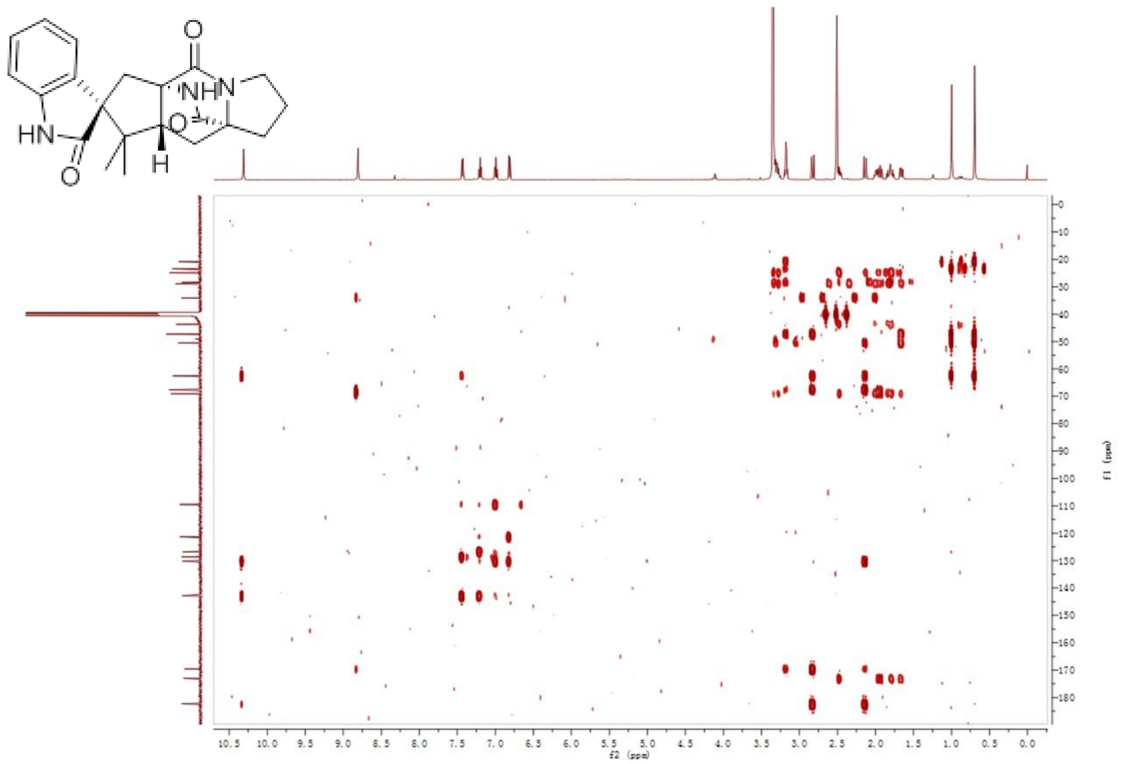

Figure S12. NOESY spectrum for compound **2**

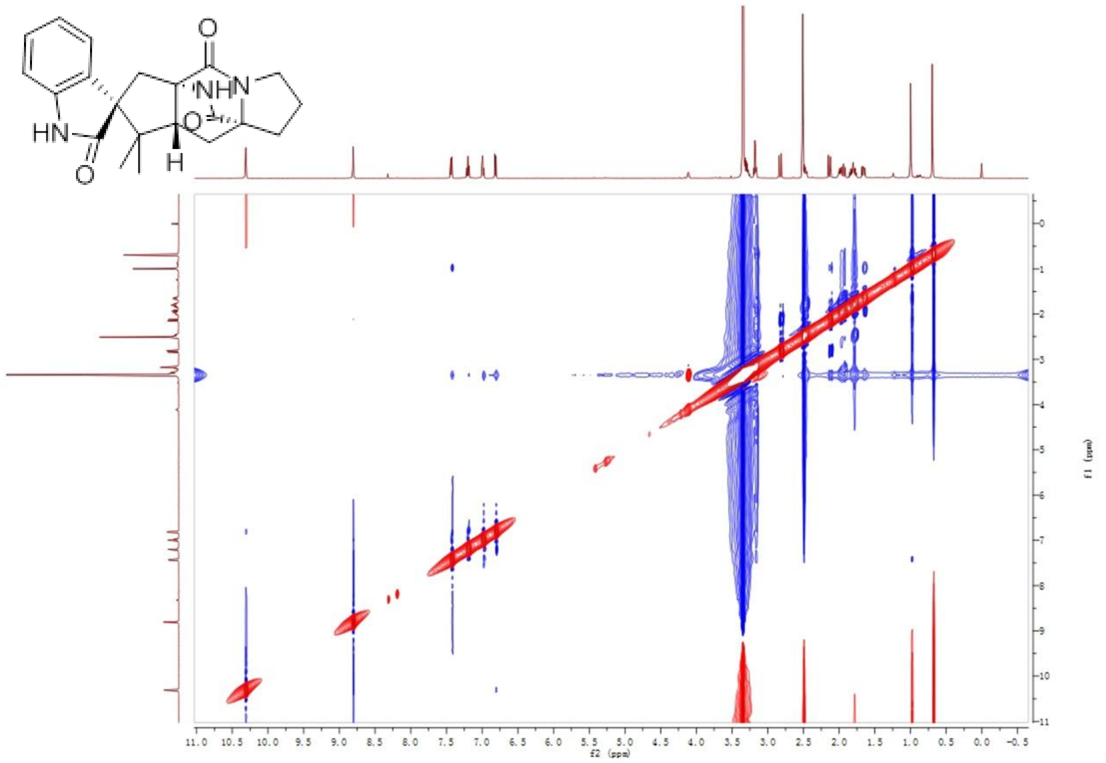

Figure S13. HR-ESIMS spectrum for compound **2**

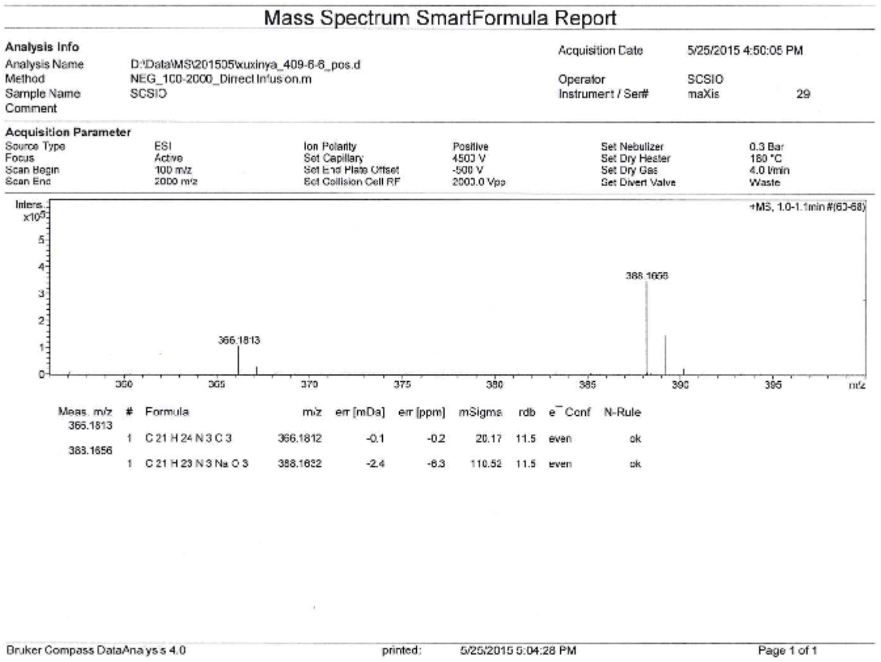

Figure S14. ^{1}H NMR spectrum for compound **3**

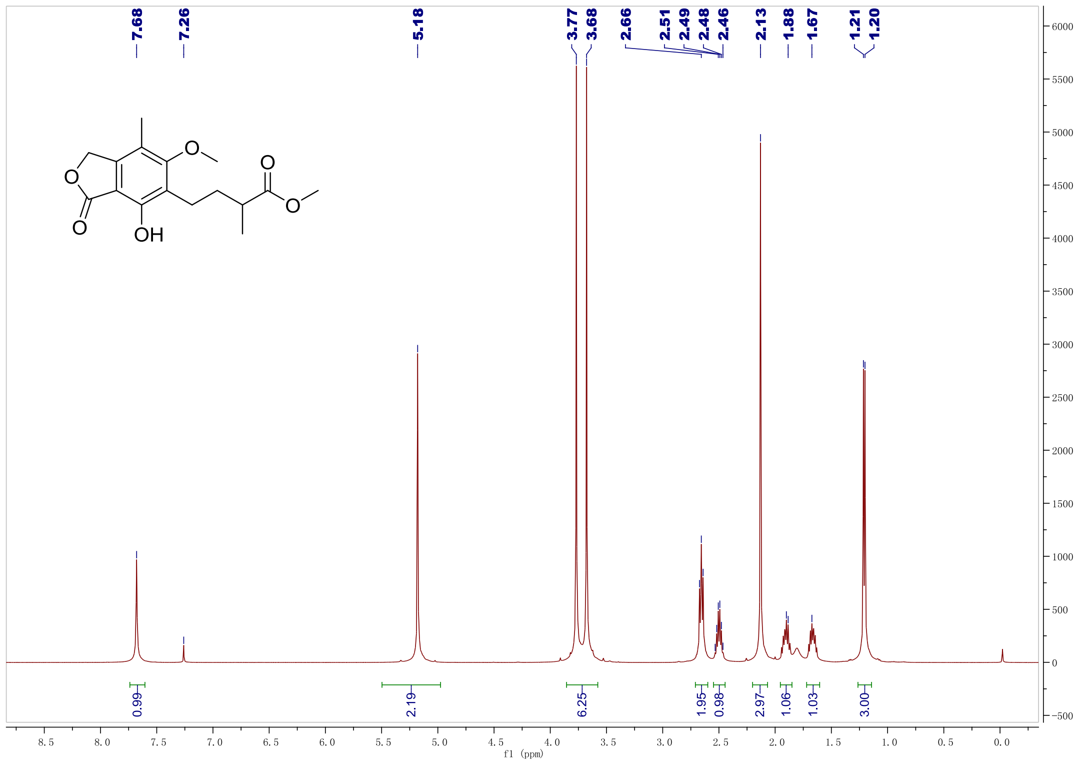

Figure S15. ^{13}C NMR spectrum for compound **3**

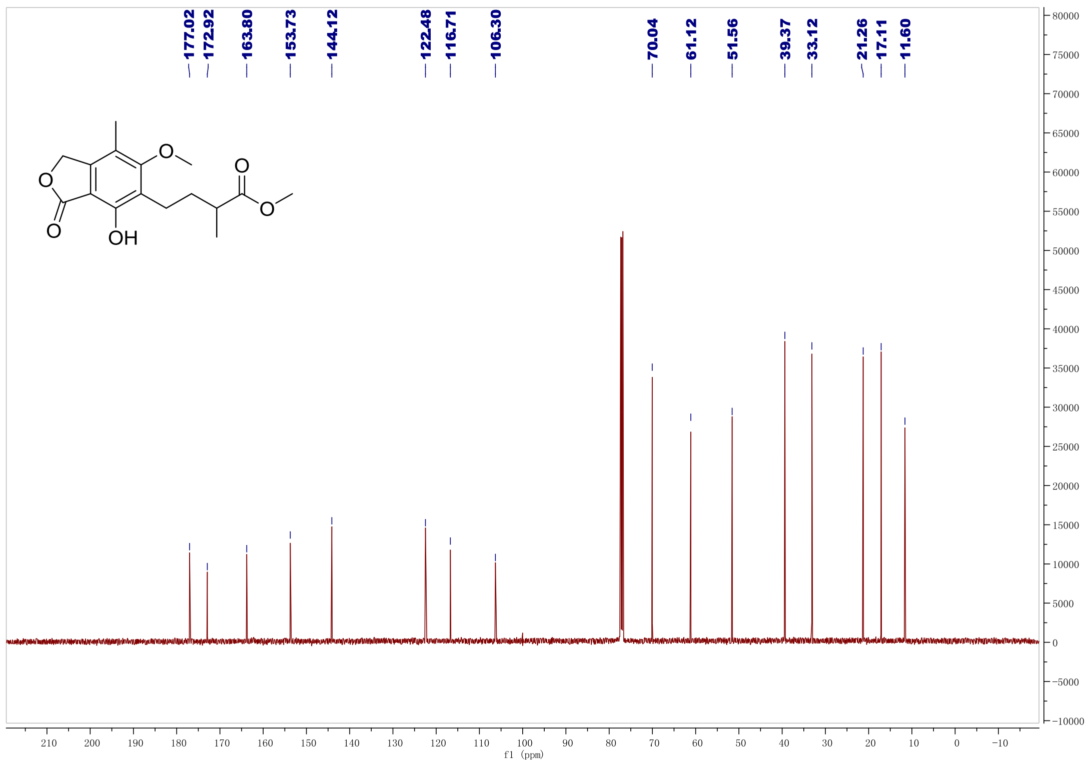

Figure S16. HSQC spectrum for compound **3**

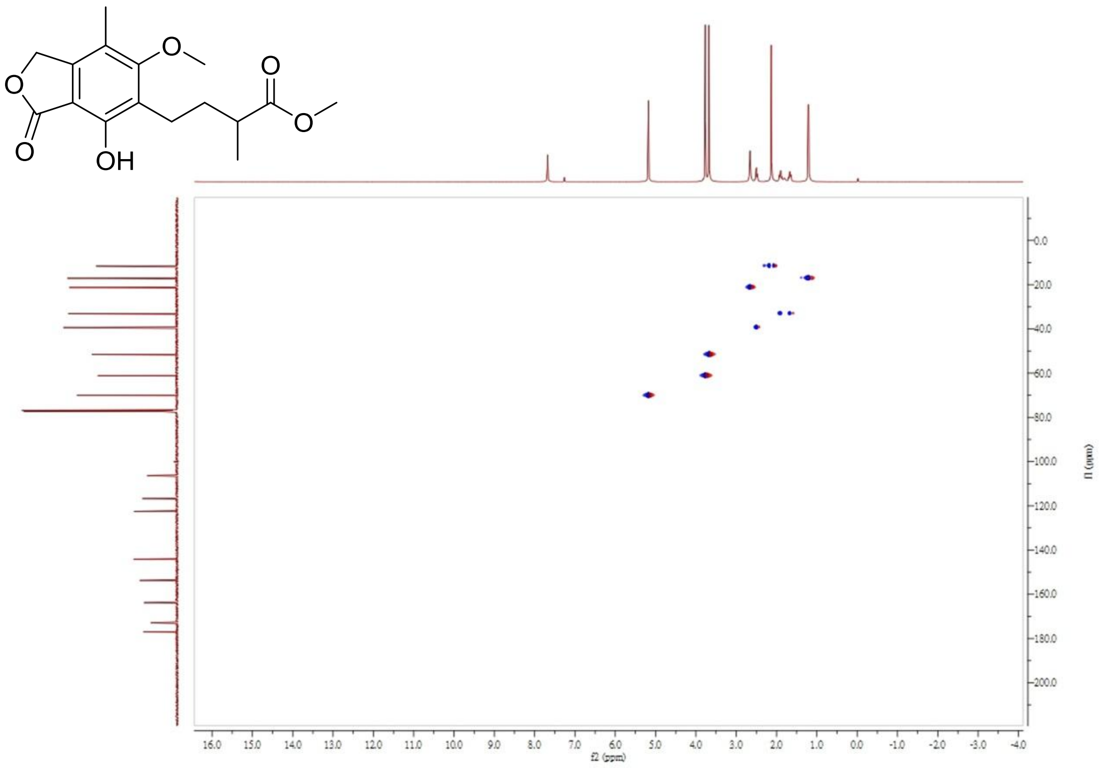

Figure S17. HMBC spectrum for compound **3**

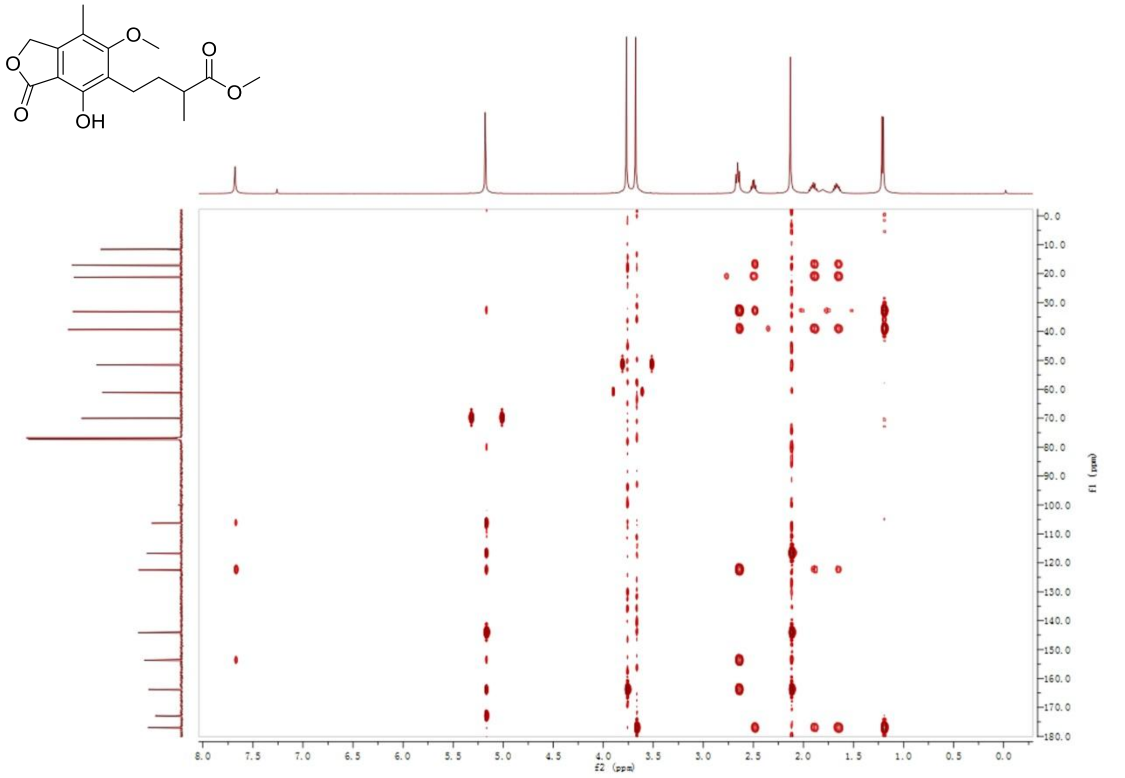

Figure S18. HR-ESIMS spectrum for compound **3**

Mass Spectrum SmartFormula Report

Analysis Info

Analysis Name	D:\Data\MS\data\2014\12\xuxinya_409-6-4_pos.d	Acquisition Date	12/29/2014 4:53:28 PM
Method	POS_100-2000_Dirrect Infusion.m	Operator	SCSIO
Sample Name	SCSIO	Instrument / Ser#	maXis 29
Comment			

Acquisition Parameter

Source Type	ESI	Ion Polarity	Positive	Set Nebulizer	0.3 Bar
Focus	Not active	Set Capillary	4500 V	Set Dry Heater	180 °C
Scan Begin	100 m/z	Set End Plate Offset	-500 V	Set Dry Gas	4.0 l/min
Scan End	2000 m/z	Set Collision Cell RF	2000.0 Vpp	Set Divert Valve	Waste

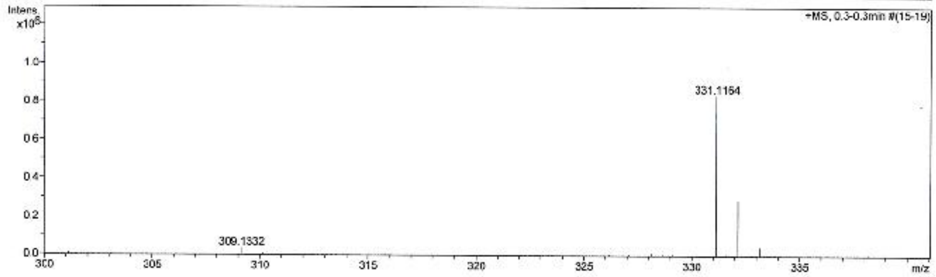

Meas. m/z	#	Formula	m/z	err [ppm]	Mean err [ppm]	rdb	N-Rule	e⁻ Conf	mSigma	Std I	Std Mean m/z	Std I VarNorm	Std m/z Diff	Std Comb Dev
309.1332	1	C 16 H 21 O 6	309.1333	0.2	0.6	6.5	ok	even	13.5	24.4	0.7	10.2	0.2	842.7
331.1164	1	C 16 H 20 Na O 6	331.1152	-3.5	-2.8	6.5	ok	even	97.8	152.9	1.0	63.5	0.6	919.7
639.2425	1	C 32 H 40 Na O 12	639.2412	-2.1	-1.7	12.5	ok	even	7.4	13.6	1.2	5.1	1.4	761.4

Bruker Compass DataAnalysis 4.0 printed: 12/29/2014 5:04:10 PM Page 1 of 1

Figure S19. ^{1}H NMR spectrum for compound **4**

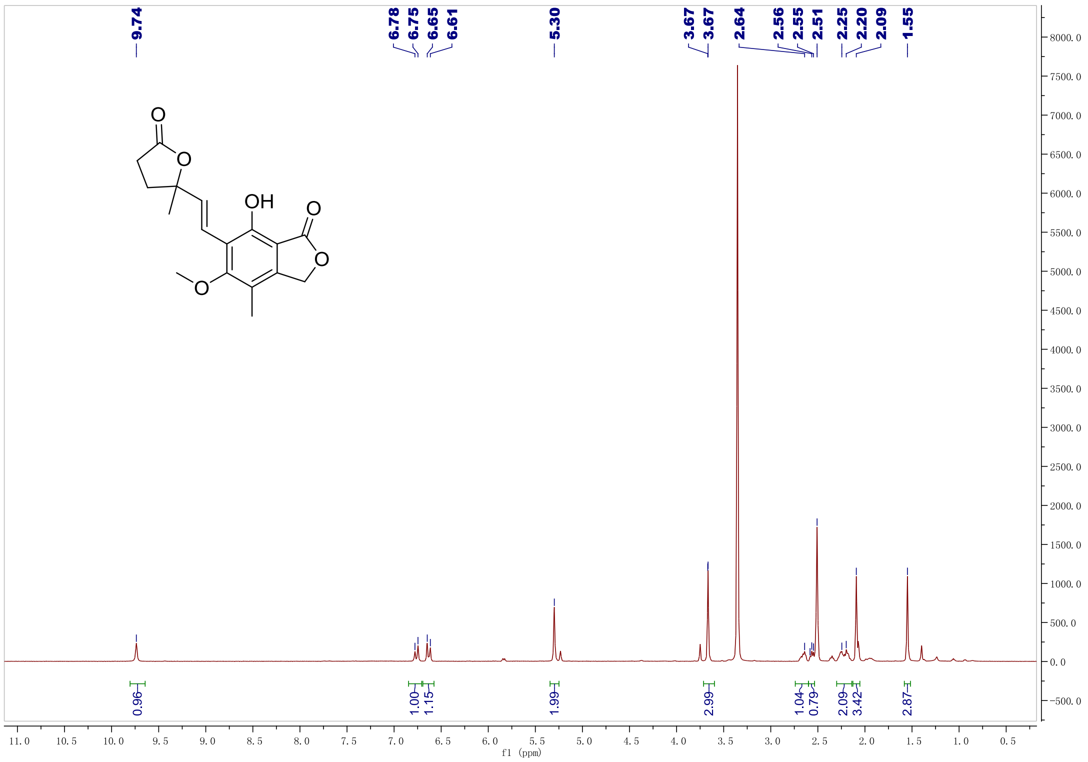

Figure S20. ^{13}C NMR spectrum for compound **4**

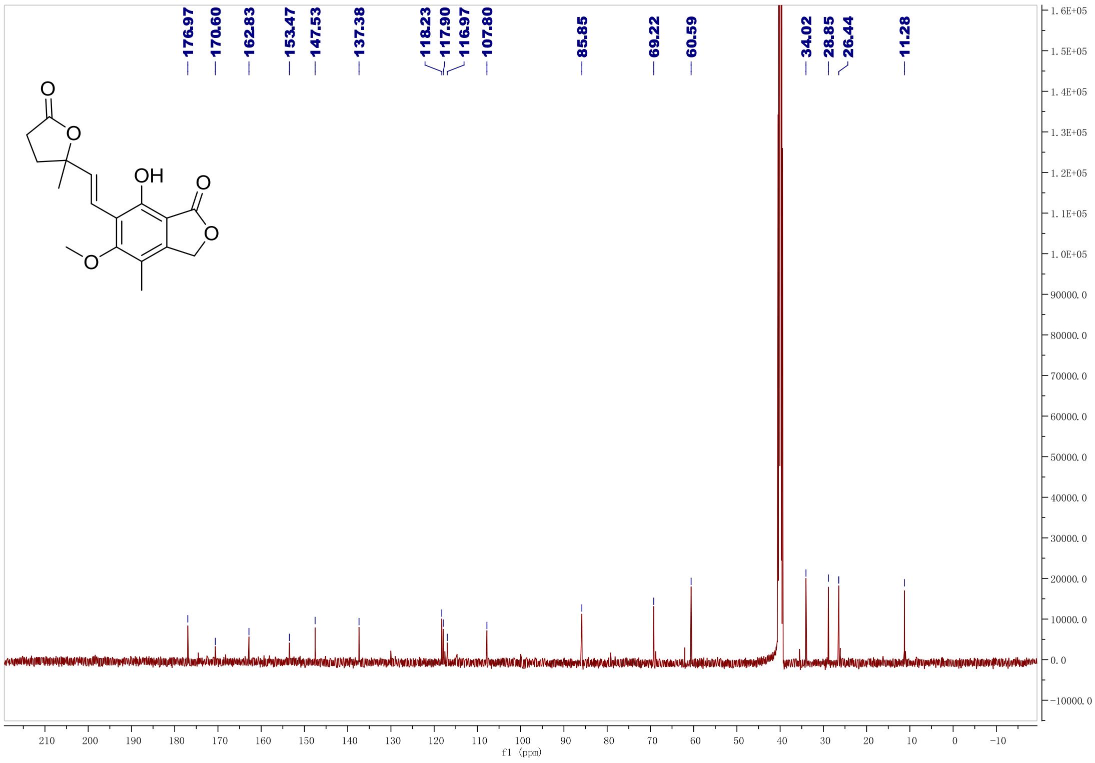

Figure S21. HSQC spectrum for compound **4**

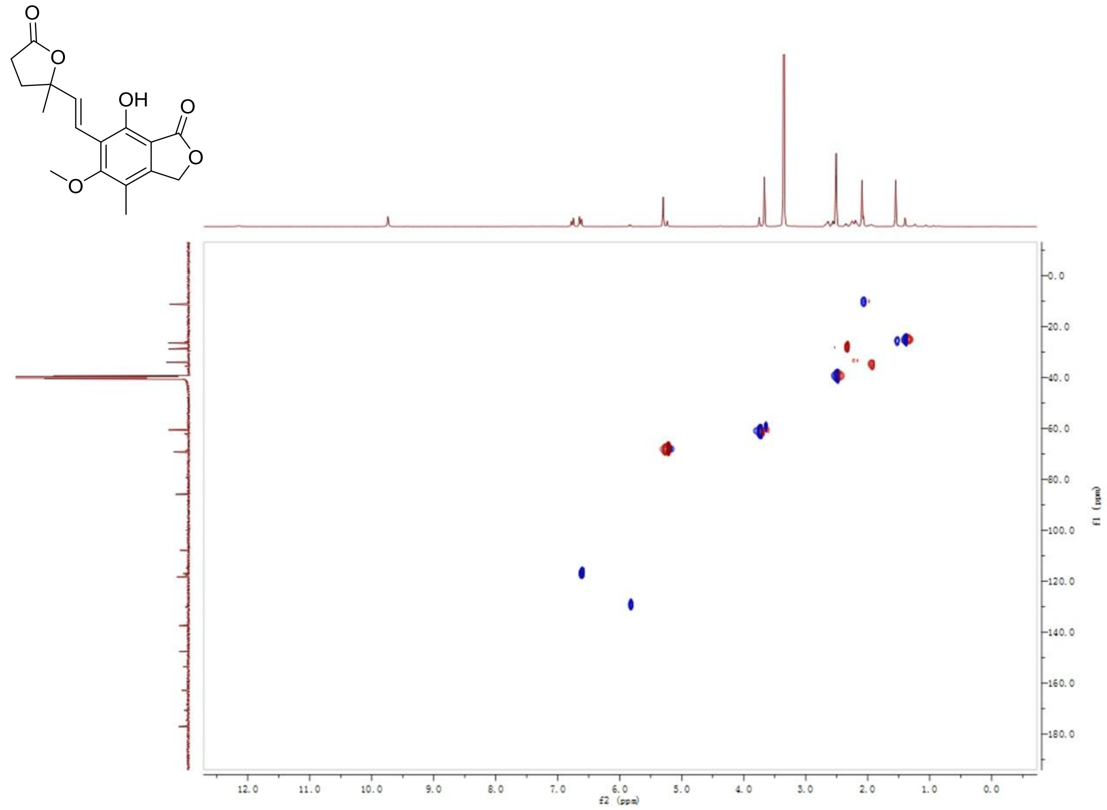

Figure S22. HMBC spectrum for compound **4**

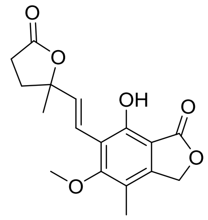

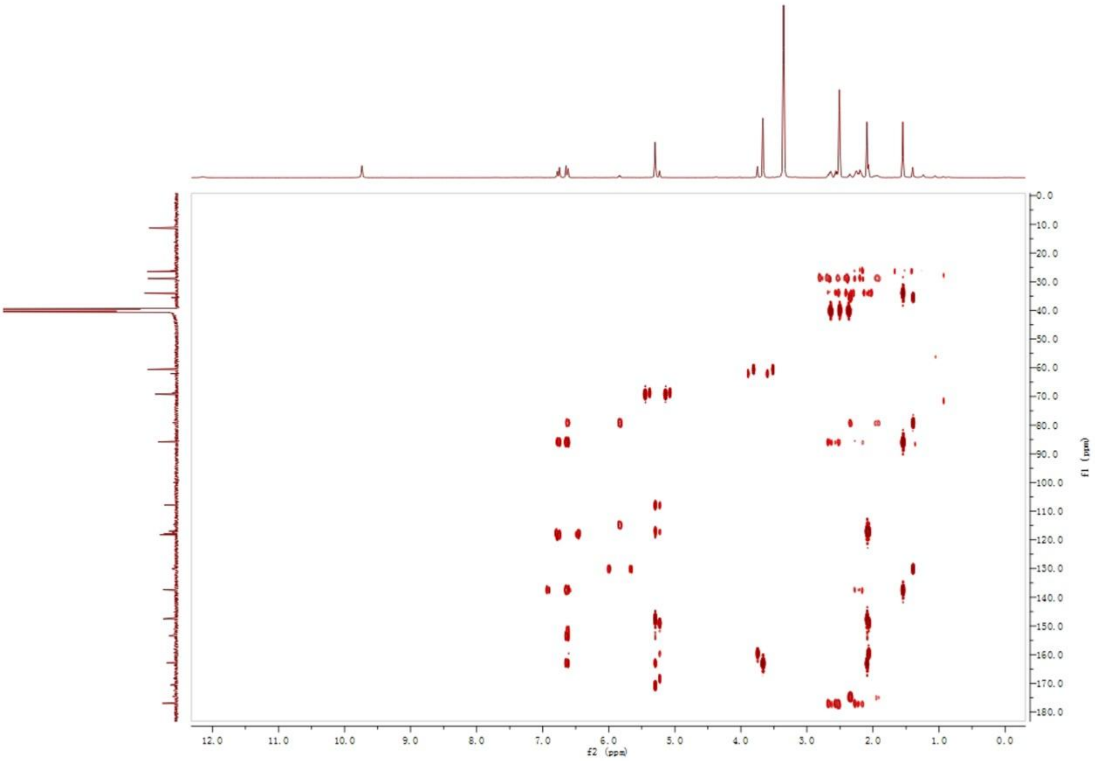

Figure S23. HR-ESIMS spectrum for compound **4**

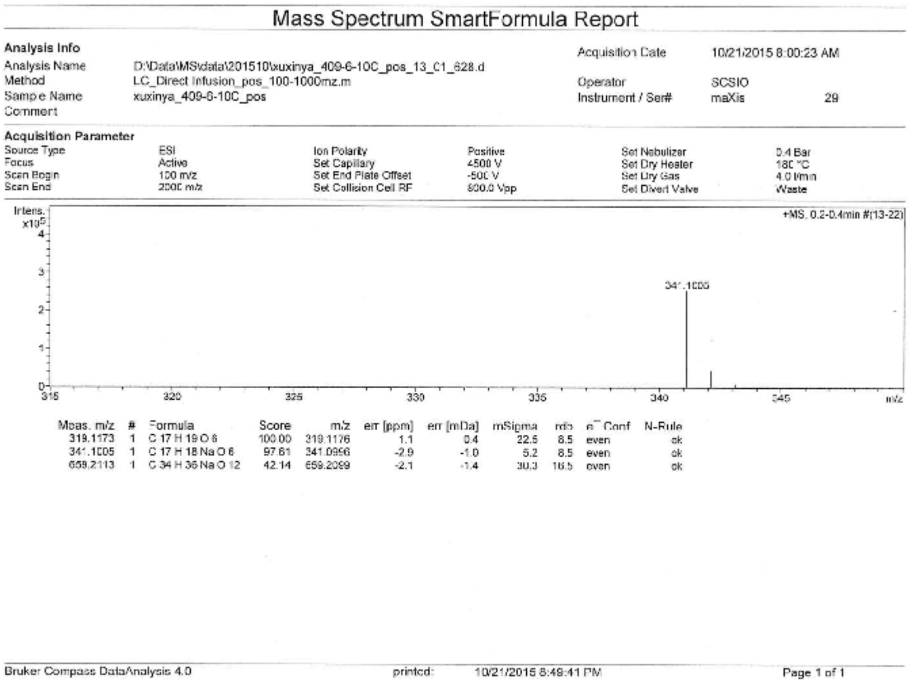

Mass Spectrum SmartFormula Report

Analysis Info

Analysis Name: D:\Data\MS\data\201510\xuxinya_409-6-10C_pos_13_C1_628.d
Method: LC_Direct Infusion_pos_100-1000mz.m
Sample Name: xuxinya_409-6-10C_pos
Comment
Acquisition Date: 10/21/2015 8:00:23 AM
Operator: SCSIO
Instrument / Ser#: maXis 29

Acquisition Parameter

Source Type	ESI	Ion Polarity	Positive	Set Nebulizer	0.4 Bar
Focus	Active	Set Capillary	4500 V	Set Dry Heater	180 °C
Scan Begin	100 m/z	Set End Plate Offset	-500 V	Set Dry Gas	4.0 l/min
Scan End	2000 m/z	Set Collision Cell RF	800.0 Vpp	Set Divert Valve	Waste

Meas. m/z	#	Formula	Score	m/z	err [ppm]	err [mDa]	mSigma	rdb	e⁻ Conf	N-Rule
319.1173	1	C 17 H 19 O 6	100.00	319.1176	1.1	0.4	22.5	8.5	even	ok
341.1005	1	C 17 H 18 Na O 6	97.61	341.0996	-2.9	-1.0	5.2	8.5	even	ok
659.2113	1	C 34 H 36 Na O 12	42.14	659.2099	-2.1	-1.4	30.3	16.5	even	ok

Bruker Compass DataAnalysis 4.0 printed: 10/21/2015 8:49:41 PM Page 1 of 1

Figure S24. ^{1}H NMR spectrum for compound **6**

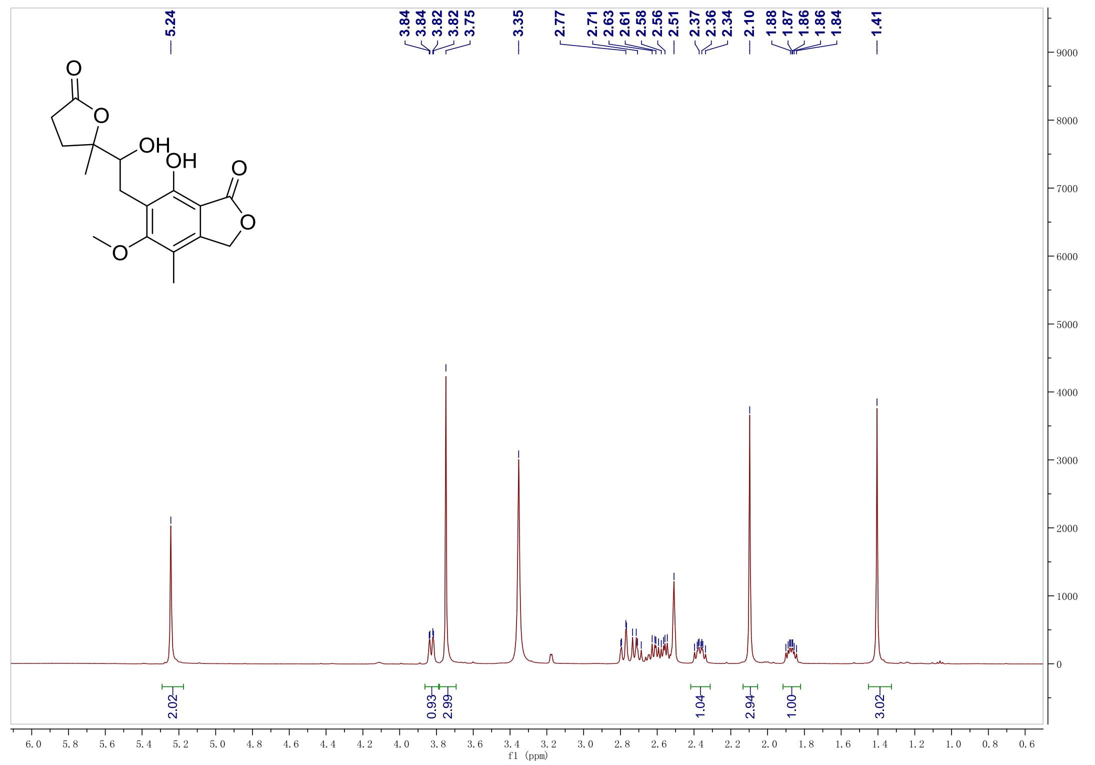

Figure S25. ^{13}C NMR spectrum for compound **6**

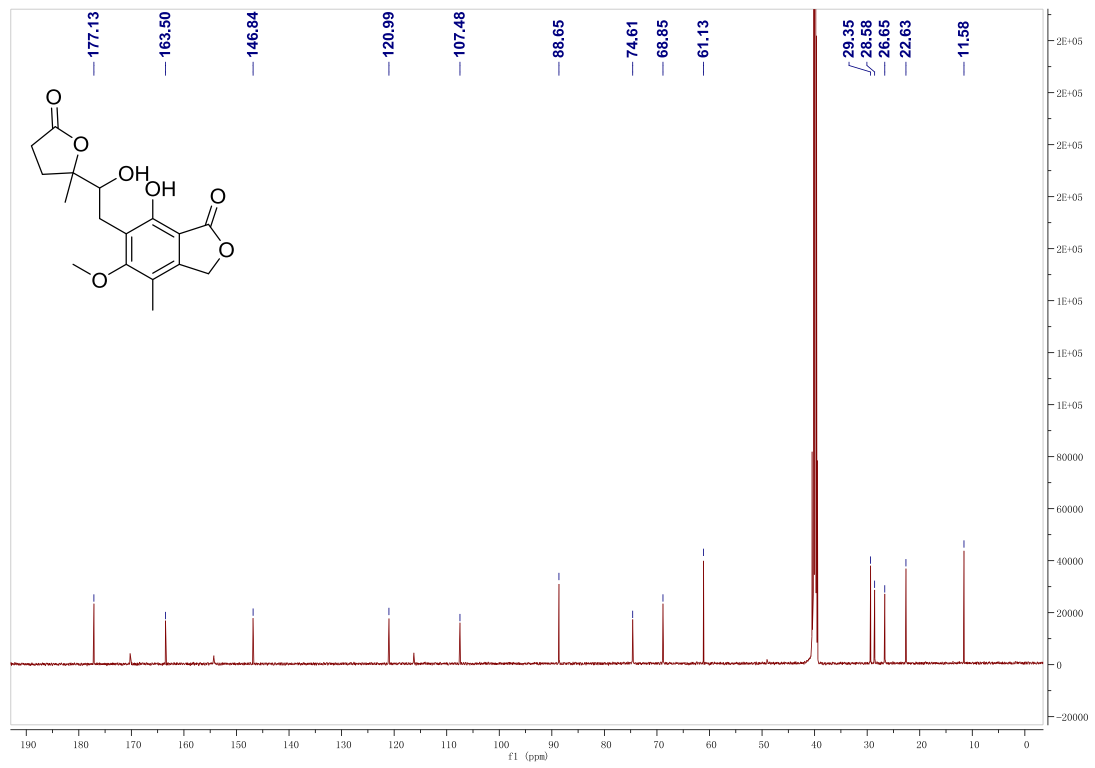

Figure S26. HSQC spectrum for compound **6**

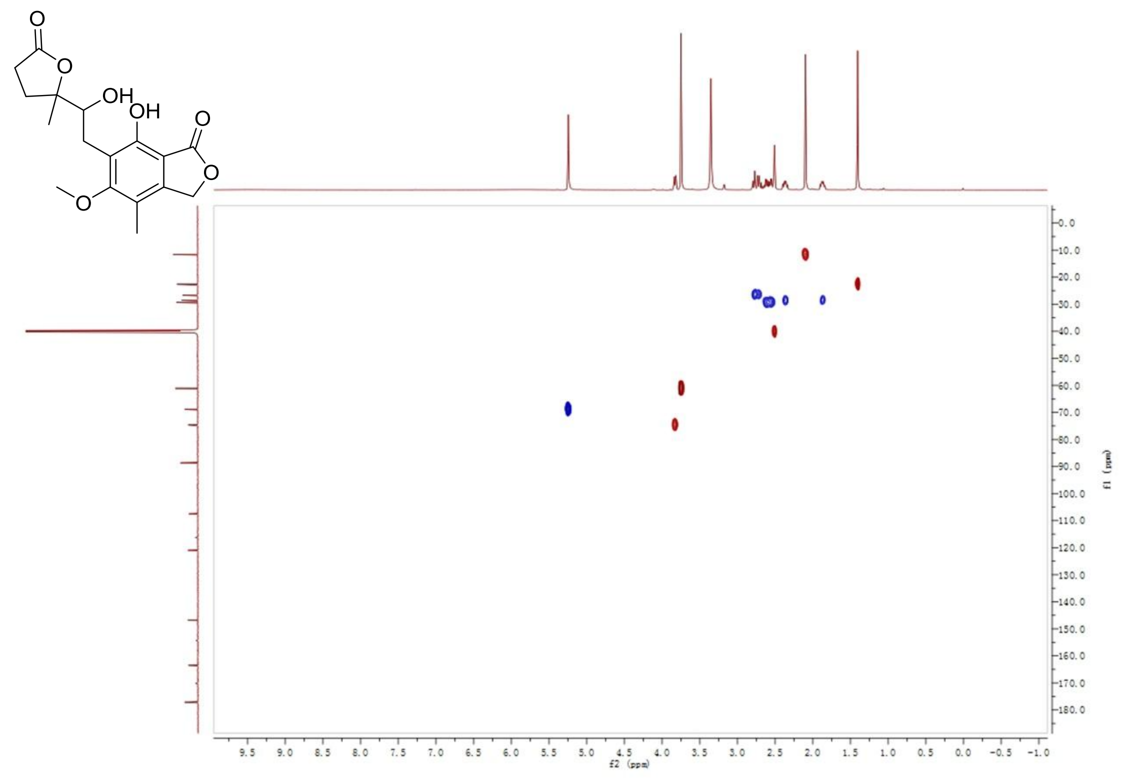

Figure S27. HMBC spectrum for compound **6**

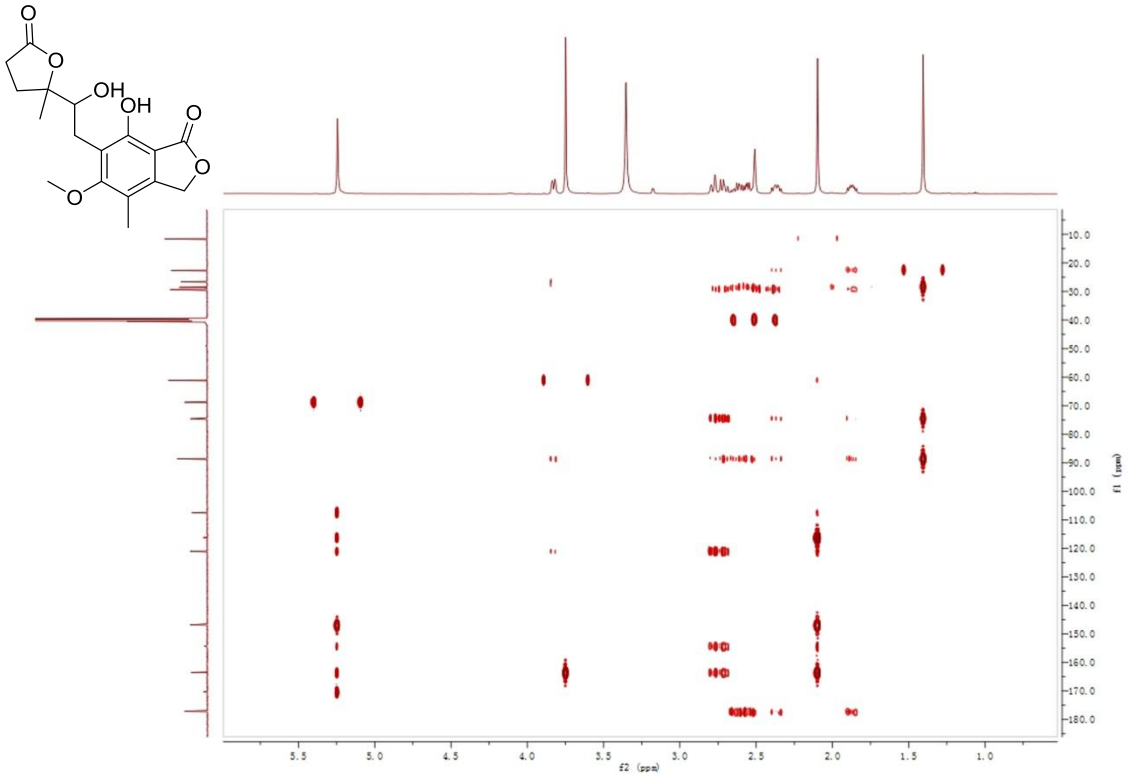

Figure S28. HR-ESIMS spectrum for compound **6**.

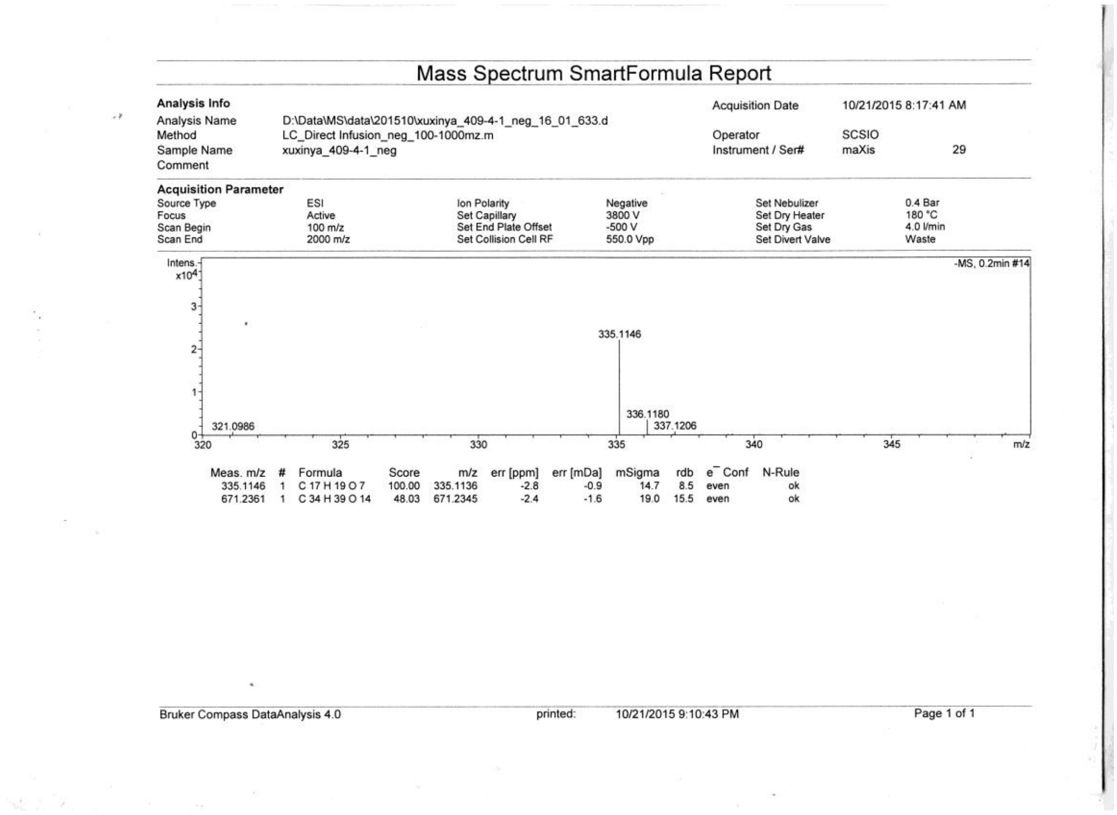

Mass Spectrum SmartFormula Report

Analysis Info

Analysis Name	D:\Data\MS\data\201510\xuxinya_409-4-1_neg_16_01_633.d	Acquisition Date	10/21/2015 8:17:41 AM
Method	LC_Direct Infusion_neg_100-1000mz.m	Operator	SCSIO
Sample Name	xuxinya_409-4-1_neg	Instrument / Ser#	maXis 29
Comment			

Acquisition Parameter

Source Type	ESI	Ion Polarity	Negative	Set Nebulizer	0.4 Bar
Focus	Active	Set Capillary	3800 V	Set Dry Heater	180 °C
Scan Begin	100 m/z	Set End Plate Offset	-500 V	Set Dry Gas	4.0 l/min
Scan End	2000 m/z	Set Collision Cell RF	550.0 Vpp	Set Divert Valve	Waste

Meas. m/z	#	Formula	Score	m/z	err [ppm]	err [mDa]	mSigma	rdb	e⁻ Conf	N-Rule
335.1146	1	C 17 H 19 O 7	100.00	335.1136	-2.8	-0.9	14.7	8.5	even	ok
671.2361	1	C 34 H 39 O 14	48.03	671.2345	-2.4	-1.6	19.0	15.5	even	ok

Bruker Compass DataAnalysis 4.0 printed: 10/21/2015 9:10:43 PM Page 1 of 1

Figure S29. Original CD spectrum for compound 6 (MeOH, 200 μg/mL)

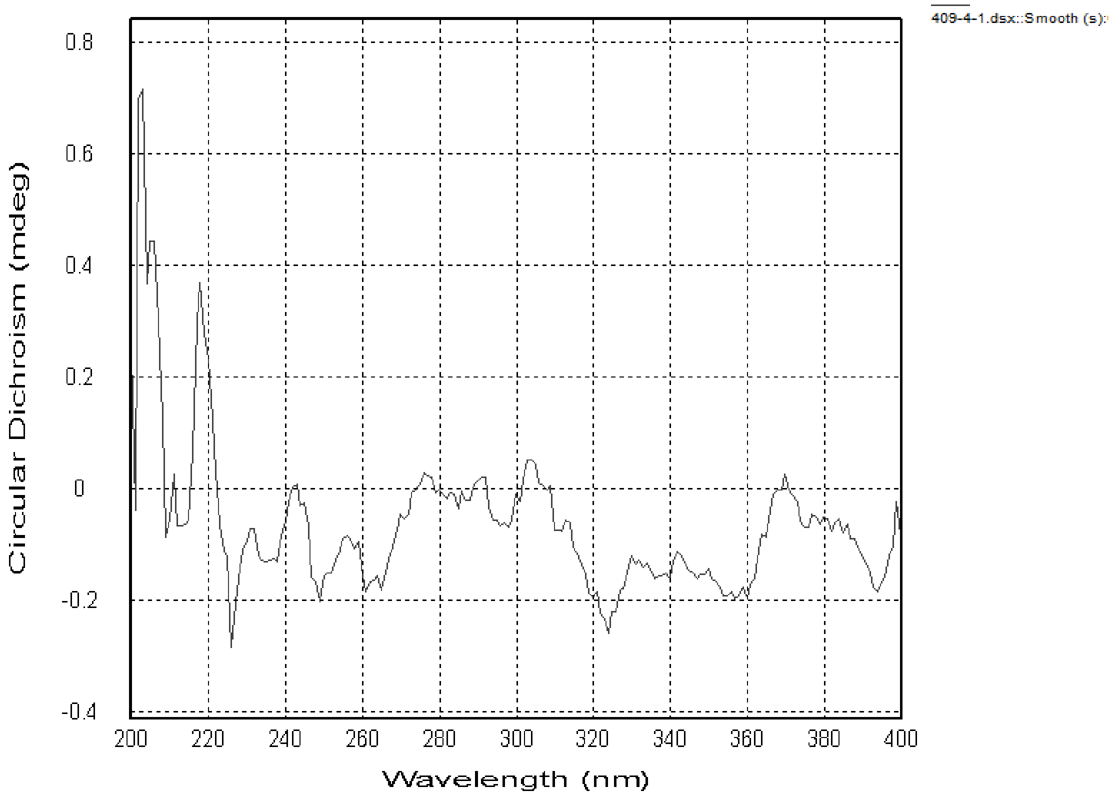

Figure S30. ^{1}H NMR spectrum for compound **8**

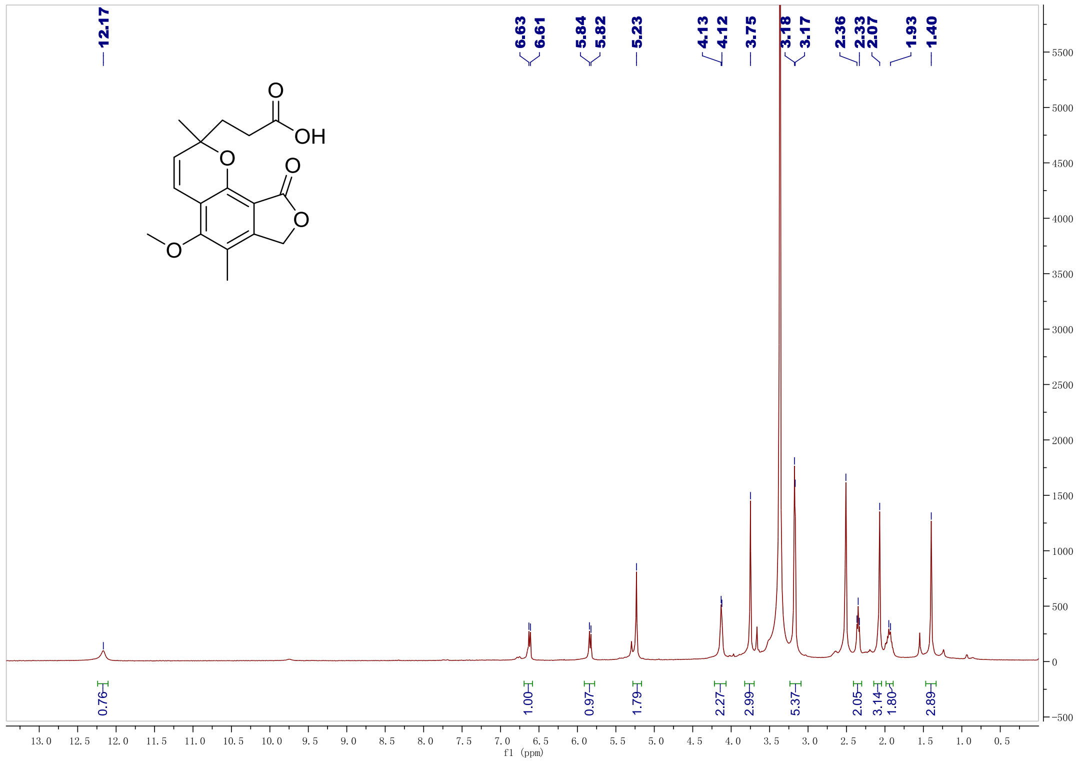

Figure S31. ^{13}C NMR spectrum for compound **8**

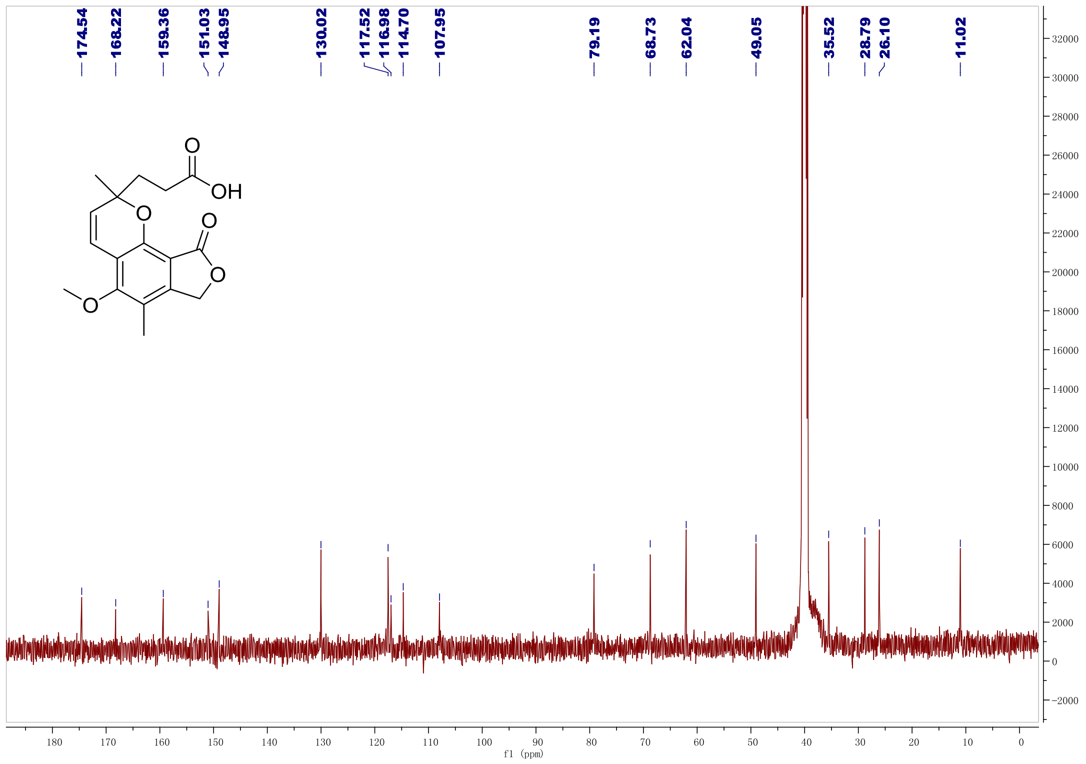

Figure S32. HMBC spectrum for compound **8**

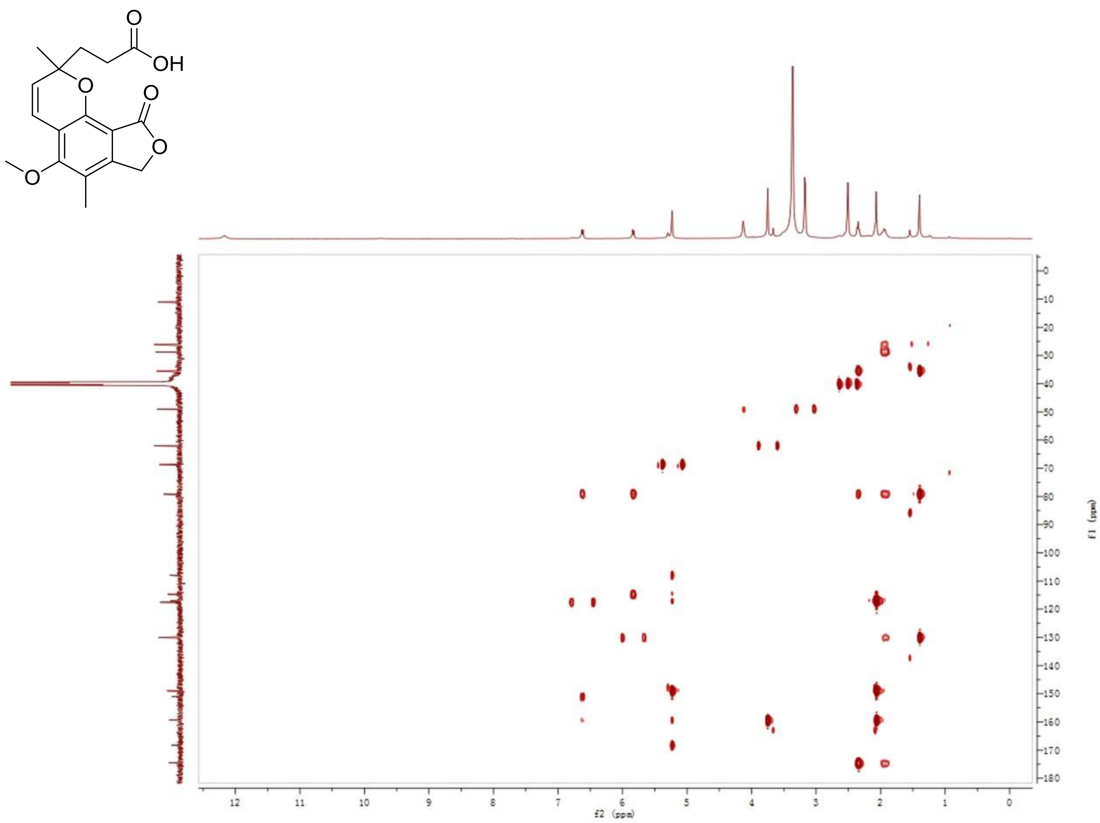

Figure S33. ESIMS spectrum for compound **8**

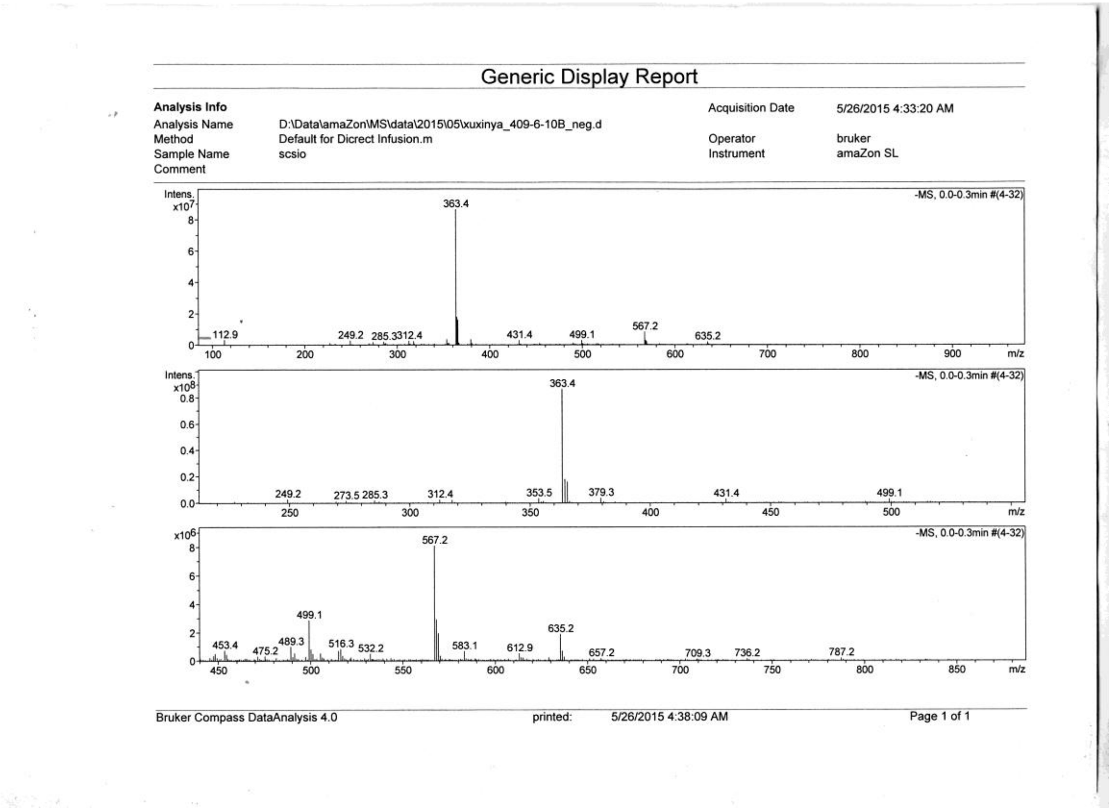

Figure S34. ^{1}H NMR spectrum for compound **9**

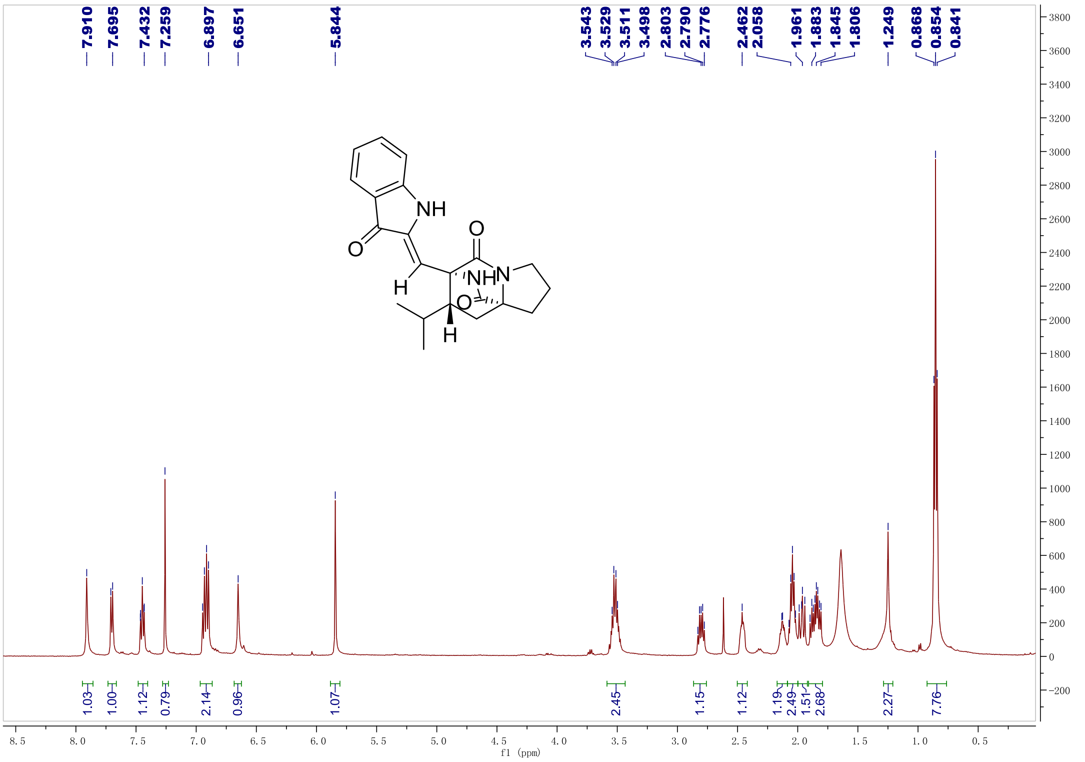

Figure S35. ^{13}C NMR spectrum for compound **9**

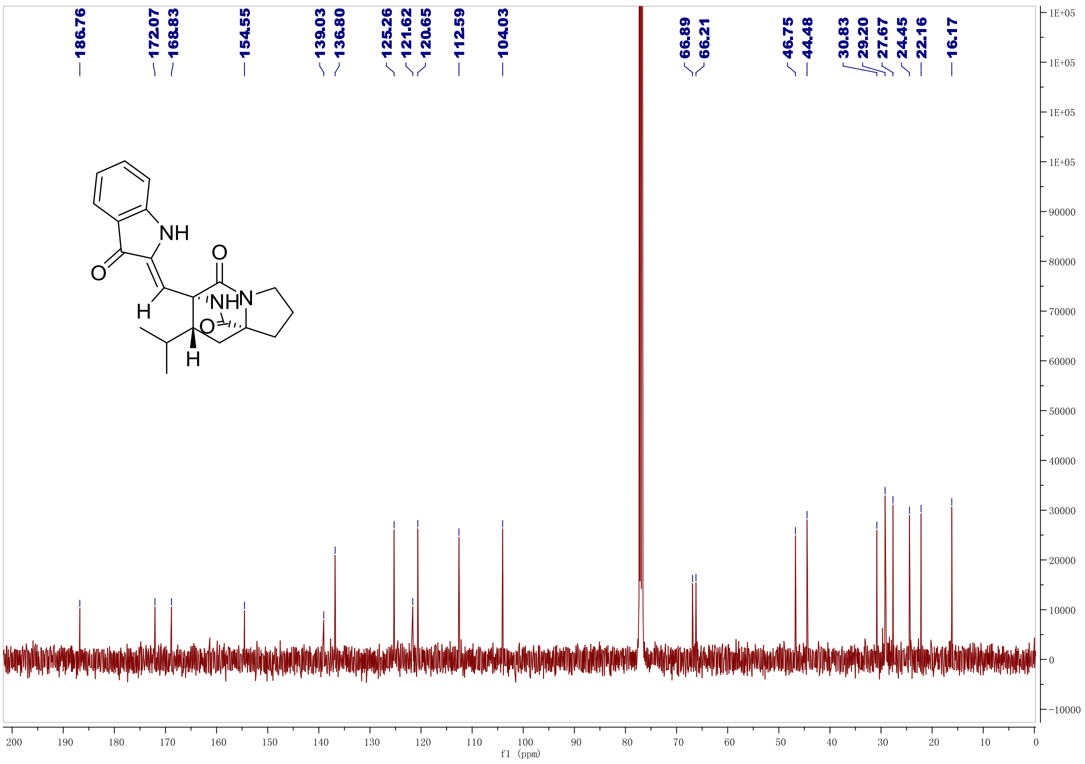

Figure S36. HSQC spectrum for compound **9**

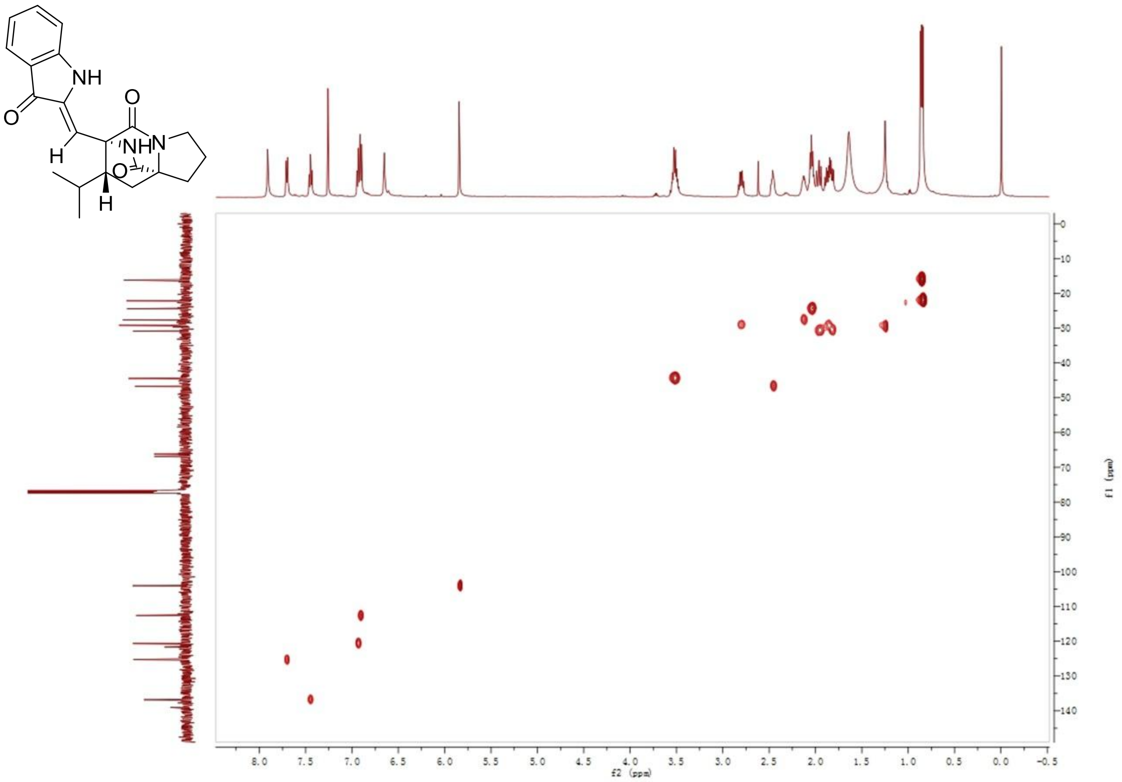

Figure S37. ^{1}H-^{1}H COSY spectrum for compound **9**

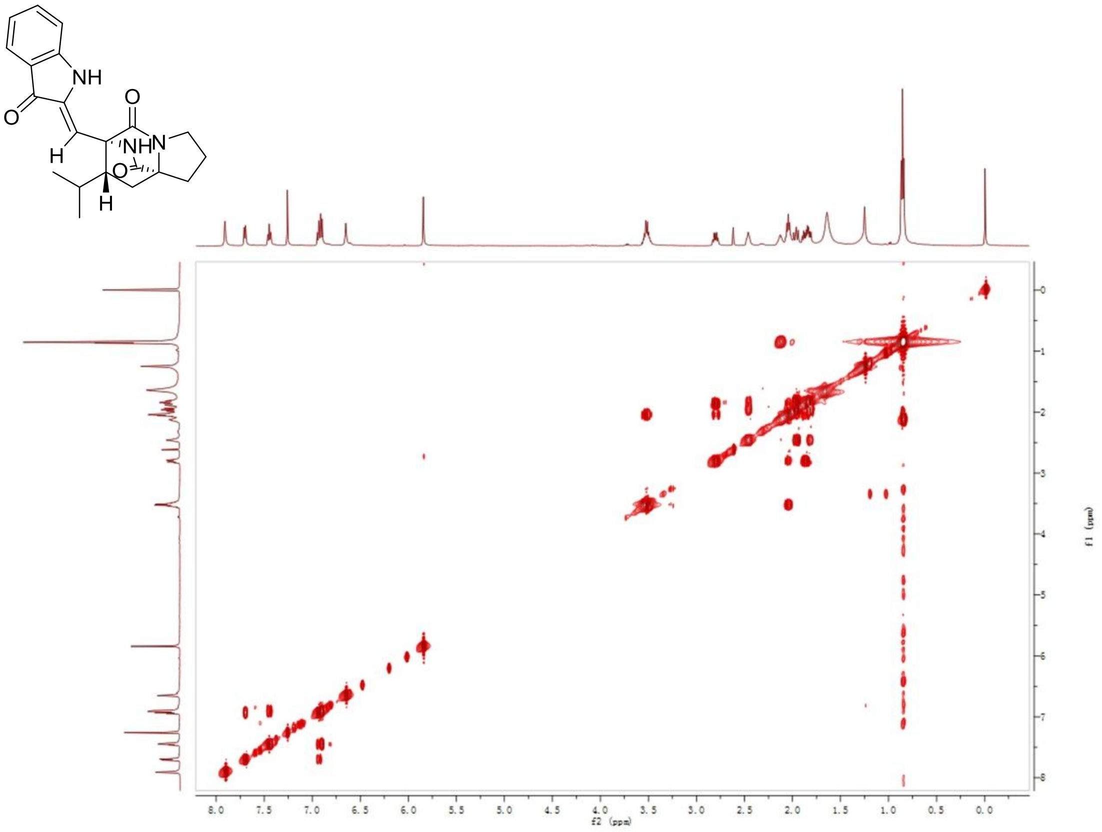

Figure S38. HMBC spectrum for compound **9**

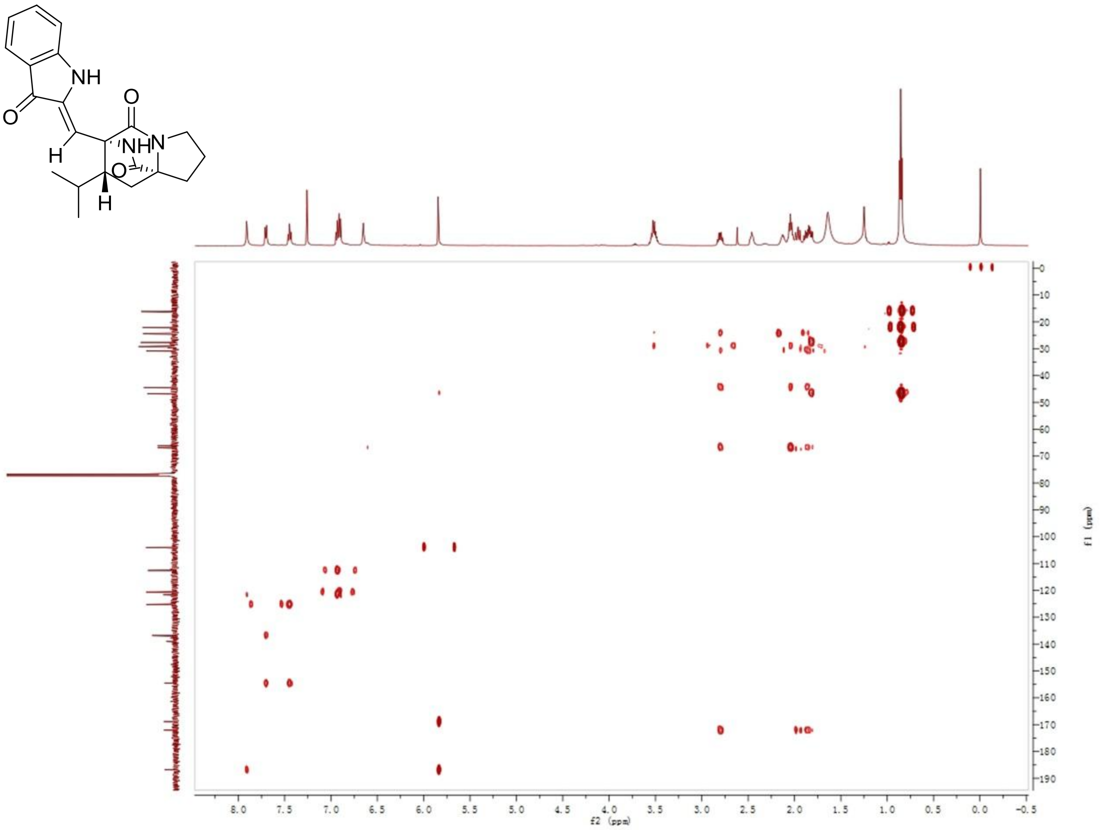

Figure S39. NOESY spectrum for compound **9**

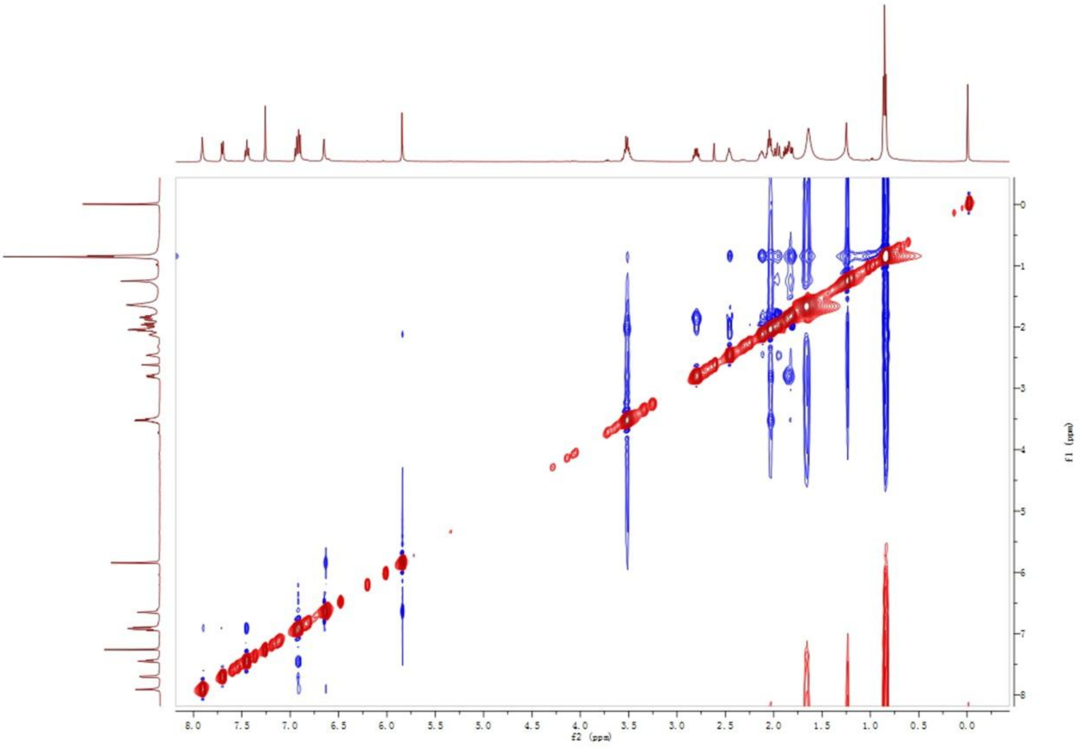

Figure S40. HR-ESIMS spectrum for compound **9**

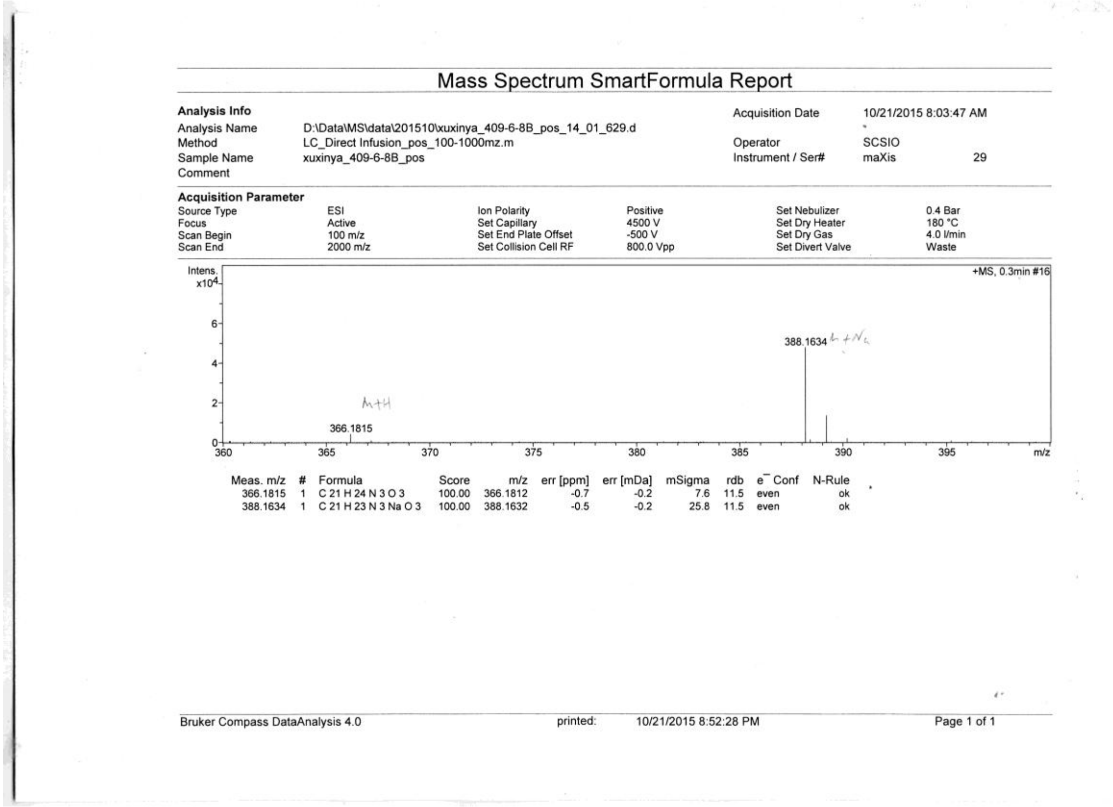

Mass Spectrum SmartFormula Report

**Analysis Info**		Acquisition Date	10/21/2015 8:03:47 AM
Analysis Name	D:\Data\MS\data\201510\xuxinya_409-6-8B_pos_14_01_629.d		
Method	LC_Direct Infusion_pos_100-1000mz.m	Operator	SCSIO
Sample Name	xuxinya_409-6-8B_pos	Instrument / Ser#	maXis 29
Comment			

Acquisition Parameter

Source Type	ESI	Ion Polarity	Positive	Set Nebulizer	0.4 Bar
Focus	Active	Set Capillary	4500 V	Set Dry Heater	180 °C
Scan Begin	100 m/z	Set End Plate Offset	-500 V	Set Dry Gas	4.0 l/min
Scan End	2000 m/z	Set Collision Cell RF	800.0 Vpp	Set Divert Valve	Waste

Meas. m/z	#	Formula	Score	m/z	err [ppm]	err [mDa]	mSigma	rdb	e⁻ Conf	N-Rule
366.1815	1	C 21 H 24 N 3 O 3	100.00	366.1812	-0.7	-0.2	7.6	11.5	even	ok
388.1634	1	C 21 H 23 N 3 Na O 3	100.00	388.1632	-0.5	-0.2	25.8	11.5	even	ok

Bruker Compass DataAnalysis 4.0 printed: 10/21/2015 8:52:28 PM Page 1 of 1

Figure S41. HPLC chromatogram for compounds **3**. HPLC condition: Angilent 1100 HPLC system; Column: Phenomenex® SYNERG I, 150 × 4.6 mm; Mobile phase: ACN/water, v/v; Gradient: 0-35 min, 5% to 100% ACN; 35-45 min, 100% ACN; 45-50 min, 100% to 5% ACN; 50-60 min, 5% ACN; Flow rate: 1 mL/min; Detection: 254 nm; Temperature: 25 ℃).

===

Injection Date : 1/26/2017 11:48:34 AM Seq. Line : 1
Sample Name : 409-6-4 Location : Vial 33
Acq. Operator : XXY Inj : 1
Inj Volume : 2 µl
Different Inj Volume from Sequence ! Actual Inj Volume : 1 µl
Acq. Method : C:\HPCHEM\1\METHODS\XUXY~1\XU.M
Last changed : 1/26/2017 10:31:52 AM by XXY
Analysis Method : C:\HPCHEM\1\METHODS\AFRA\ANA150X4.6\10-75E.M
Last changed : 1/26/2017 10:42:08 AM by AFRA
(modified after loading)

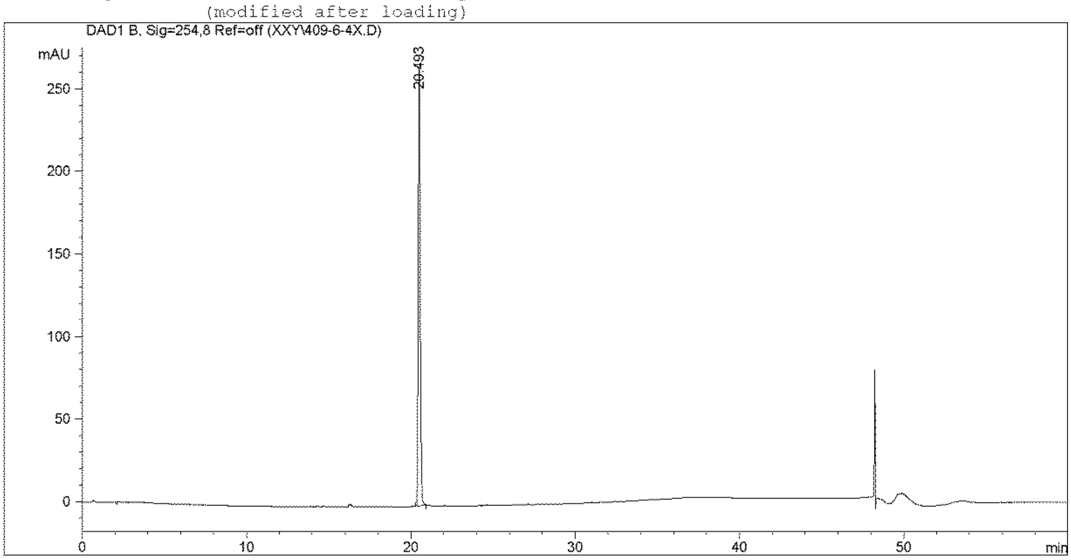

===

Area Percent Report

===

Sorted By : Signal
Multiplier : 1.0000
Dilution : 1.0000
Use Multiplier & Dilution Factor with ISTDs

Signal 1: DAD1 B, Sig=254,8 Ref=off

Peak #	RetTime [min]	Type	Width [min]	Area [mAU*s]	Height [mAU]	Area %
1	20.493	BB	0.1247	2149.82813	264.90973	100.0000

Totals : 2149.82813 264.90973

Results obtained with enhanced integrator!

===

*** End of Report ***

Figure S42. HPLC chromatogram for compound **5**. HPLC condition: same with S41.

===

Injection Date : 1/26/2017 10:34:23 AM Seq. Line : 1
Sample Name : 409-6-2 Location : Vial 32
Acq. Operator : XXY Inj : 1
Inj Volume : 2 µl
Acq. Method : C:\HPCHEM\1\METHODS\XUXY~1\XU.M
Last changed : 1/26/2017 10:31:52 AM by XXY
Analysis Method : C:\HPCHEM\1\METHODS\AFRA\ANA150X4.6\10-75E.M
Last changed : 1/26/2017 10:42:08 AM by AFRA
(modified after loading)

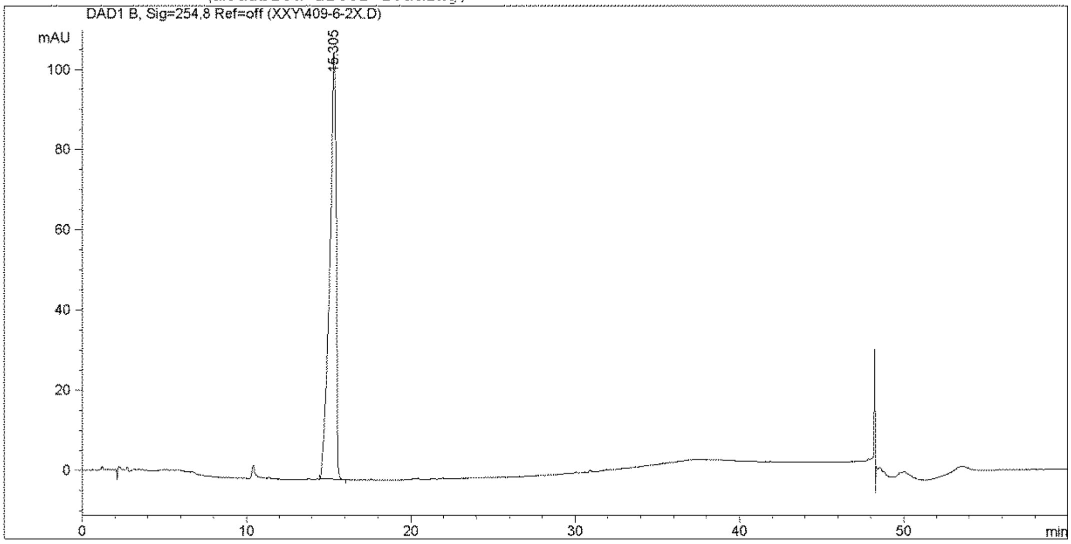

===

Area Percent Report

===

Sorted By : Signal
Multiplier : 1.0000
Dilution : 1.0000
Use Multiplier & Dilution Factor with ISTDs

Signal 1: DAD1 B, Sig=254,8 Ref=off

Peak #	RetTime [min]	Type	Width [min]	Area [mAU*s]	Height [mAU]	Area %
1	15.305	BB	0.3845	2909.28076	106.56707	100.0000

Totals : 2909.28076 106.56707

Results obtained with enhanced integrator!

===

*** End of Report ***

Figure S43. Chiral HPLC chromatogram spectrum for compound **3**. HPLC condition: Angilent 1100 HPLC system; Column: CHIRALPAK® IC, 250 × 4.6 mm; Mobile

phase: n-hexane/propan-2-ol 70:30, v/v; Flow rate: 1 mL/min; Detection: 254 nm; Temperature: 25 ℃.

Data File C:\HPCHEM\1\DATA\XXY\6-4CHIR5.D Sample Name: 406-6-4

```
=====================================================================
Injection Date  : 1/27/2017 1:06:45 AM          Seq. Line :   1
Sample Name     : 406-6-4                        Location : Vial 32
Acq. Operator   : XXY                                 Inj :   1
                                               Inj Volume : 5 µl
Acq. Method     : C:\HPCHEM\1\METHODS\XU-P.M\XU-P.M
Last changed    : 1/27/2017 1:05:50 AM by XXY
Analysis Method : C:\HPCHEM\1\METHODS\AFRA\ANA150X4.6\10-75E.M
Last changed    : 1/26/2017 11:12:39 PM by AFRA
                  (modified after loading)
```

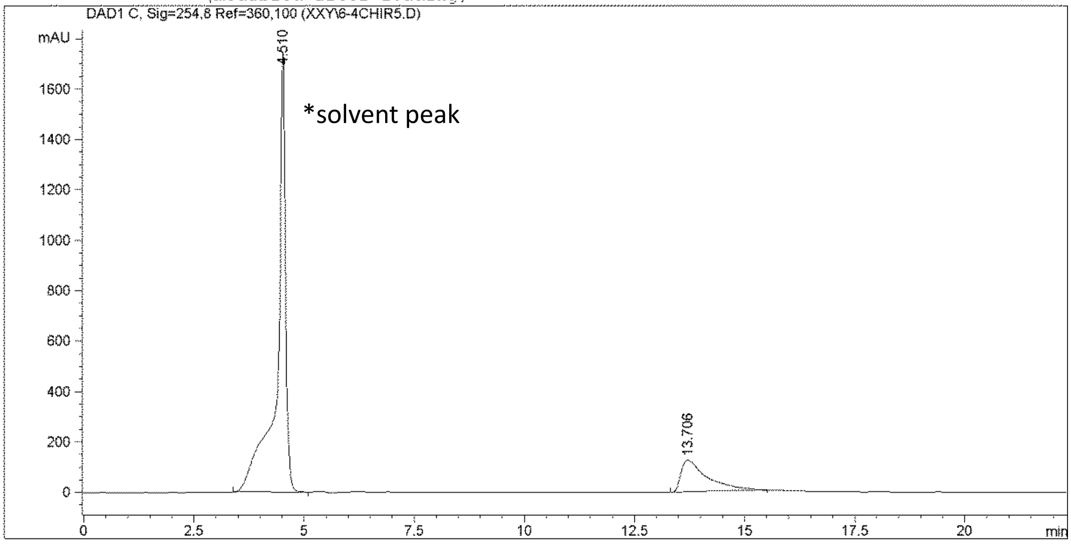


```
=====================================================================
                         Area Percent Report
=====================================================================

Sorted By             :      Signal
Multiplier            :      1.0000
Dilution              :      1.0000
Use Multiplier & Dilution Factor with ISTDs


Signal 1: DAD1 C, Sig=254,8 Ref=360,100

Peak RetTime Type  Width     Area      Height     Area
  #   [min]        [min]   [mAU*s]     [mAU]        %
----|-------|----|-------|----------|----------|--------|
   1   4.510 BV    0.1842 2.37266e4  1749.99597  81.7529
   2  13.706 PB    0.6055 5295.71484  125.20427  18.2471

Totals :                  2.90223e4  1875.20024

 Results obtained with enhanced integrator!
=====================================================================
                         *** End of Report ***
```


Figure S44. Chiral HPLC chromatogram spectrum for compound **6**. HPLC condition: Angilent 1100 HPLC system; Column: CHIRALPAK® IC, 250 × 4.6 mm; Mobile

phase: n-hexane/propan-2-ol 60:40, v/v; Flow rate: 1 mL/min; Detection: 254 nm; Temperature: 25 ℃).


```
=====================================================================
Injection Date  : 1/27/2017 12:26:17 AM          Seq. Line :    1
Sample Name     : 409-4-1                          Location : Vial 34
Acq. Operator   : XXY                                   Inj :    1
                                                Inj Volume : 5 µl
Different Inj Volume from Sequence !     Actual Inj Volume : 2 µl
Acq. Method     : C:\HPCHEM\1\METHODS\XU-P.M\XU-P.M
Last changed    : 1/26/2017 11:59:52 PM by XXY
Analysis Method : C:\HPCHEM\1\METHODS\AFRA\ANA150X4.6\10-75E.M
Last changed    : 1/26/2017 11:12:39 PM by AFRA
                  (modified after loading)
```

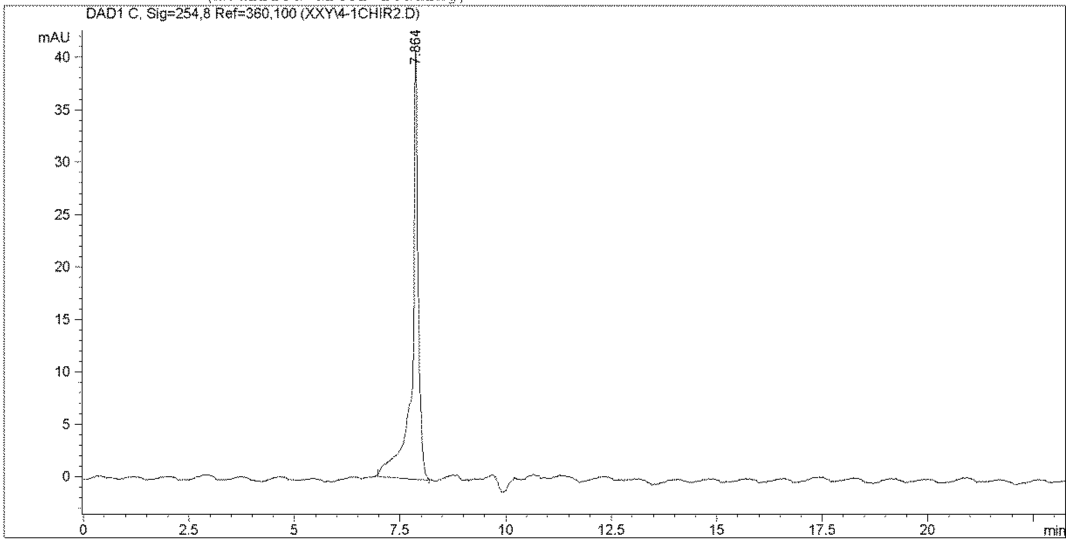


```
=====================================================================
                         Area Percent Report
=====================================================================

Sorted By             :      Signal
Multiplier            :      1.0000
Dilution              :      1.0000
Use Multiplier & Dilution Factor with ISTDs


Signal 1: DAD1 C, Sig=254,8 Ref=360,100
```

Peak #	RetTime [min]	Type	Width [min]	Area [mAU*s]	Height [mAU]	Area %
1	7.864	BB	0.1342	414.31332	40.89666	100.0000

Totals : 414.31332 40.89666

Results obtained with enhanced integrator!

```
=====================================================================
                         *** End of Report ***
```
